# Environmental and geographical factors influencing the spread of SARS-CoV-2 over 2 years: a fine-scale spatiotemporal analysis

**DOI:** 10.3389/fpubh.2024.1298177

**Published:** 2024-06-18

**Authors:** David De Ridder, Anaïs Ladoy, Yangji Choi, Damien Jacot, Séverine Vuilleumier, Idris Guessous, Stéphane Joost, Gilbert Greub

**Affiliations:** ^1^Geographic Information Research and Analysis in Population Health (GIRAPH) Lab, Faculty of Medicine, University of Geneva (UNIGE), Geneva, Switzerland; ^2^Geospatial Molecular Epidemiology Group (GEOME), Laboratory for Biological Geochemistry (LGB), School of Architecture, Civil and Environmental Engineering (ENAC), École Polytechnique Fédérale de Lausanne (EPFL), Lausanne, Switzerland; ^3^Division and Department of Primary Care Medicine, Geneva University Hospitals, Geneva, Switzerland; ^4^Faculty of Medicine, University of Geneva, Geneva, Switzerland; ^5^Institute of Microbiology, Lausanne University Hospital and University of Lausanne, Lausanne, Switzerland; ^6^La Source School of Nursing, University of Applied Sciences and Arts Western Switzerland (HES-SO), Lausanne, Switzerland; ^7^Infectious Diseases Service, Lausanne University Hospital, Lausanne, Switzerland

**Keywords:** SARS-CoV-2, sociodemographic and environmental determinants, air pollution, spatial modeling, machine learning, geoAI, remote sensing, spatial epidemiology

## Abstract

**Introduction:**

Since its emergence in late 2019, the SARS-CoV-2 virus has led to a global health crisis, affecting millions and reshaping societies and economies worldwide. Investigating the determinants of SARS-CoV-2 diffusion and their spatiotemporal dynamics at high spatial resolution is critical for public health and policymaking.

**Methods:**

This study analyses 194,682 georeferenced SARS-CoV-2 RT-PCR tests from March 2020 and April 2022 in the canton of Vaud, Switzerland. We characterized five distinct pandemic periods using metrics of spatial and temporal clustering like inverse Shannon entropy, the Hoover index, Lloyd’s index of mean crowding, and the modified space–time DBSCAN algorithm. We assessed the demographic, socioeconomic, and environmental factors contributing to cluster persistence during each period using eXtreme Gradient Boosting (XGBoost) and SHapley Additive exPlanations (SHAP), to consider non-linear and spatial effects.

**Results:**

Our findings reveal important variations in the spatial and temporal clustering of cases. Notably, areas with flatter epidemics had higher total attack rate. Air pollution emerged as a factor showing a consistent positive association with higher cluster persistence, substantiated by both immission models and, to a lesser extent, tropospheric NO_2_ estimations. Factors including population density, testing rates, and geographical coordinates, also showed important positive associations with higher cluster persistence. The socioeconomic index showed no significant contribution to cluster persistence, suggesting its limited role in the observed dynamics, which warrants further research.

**Discussion:**

Overall, the determinants of cluster persistence remained across the study periods. These findings highlight the need for effective air quality management strategies to mitigate air pollution’s adverse impacts on public health, particularly in the context of respiratory viral diseases like COVID-19.

## Graphical abstract

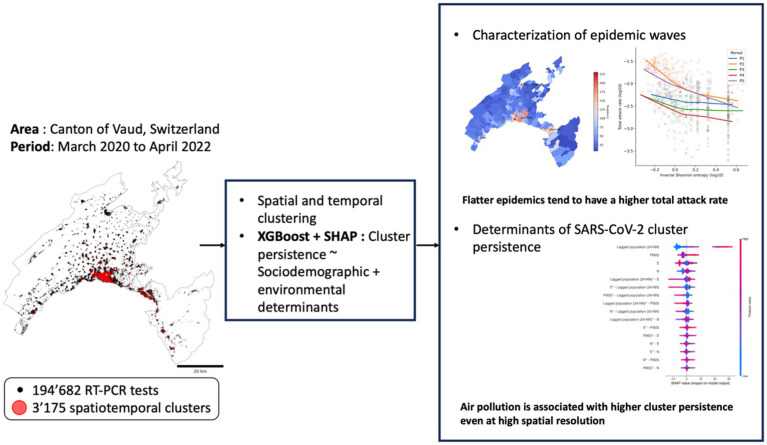

## Highlights

- High spatiotemporal resolution study of SARS-CoV-2 influencing spread over 2 years.
- Areas with flatter epidemics have higher total attack rates.
- Air pollution is positively associated with SARS-CoV-2 cluster persistence.
- No significant link between socioeconomic index and cluster persistence.
- Factors influencing SARS-CoV-2 spread are stable across periods.

## Introduction

The SARS-CoV-2 pandemic has had a significant impact on the world’s population and understanding the spatial and temporal patterns of its spread and its evolution is crucial for epidemic surveillance and control (1–5). Techniques such as hot-spot analysis, spatiotemporal clustering, and space–time scan statistics have been widely employed to analyze georeferenced data from SARS-CoV-2 RT-PCR testing (6–11). These analyses have revealed that the incidence and the mortality of the disease are not evenly distributed but rather cluster in certain areas and peak at certain times, indicating a high degree of heterogeneity in the diffusion dynamics of the virus (12–14).

To further understand the factors driving these patterns and disparities, subsequent research using methods such as regression modeling has provided better insights into the potential demographic, socioeconomic, and environmental determinants of the virus’s spread (7, 15–18). This research conducted since the beginning of the pandemic and over more than 2 years has revealed the complexity of the issue, highlighting the intricate and interconnected array of factors influencing the spread of the virus at different geographical and temporal scales.

Human mobility, connectivity, and transportation have been identified as key factors facilitating the virus’ spread (14, 19–22). Additionally, other reports have emphasized the importance of socioeconomic conditions, with socioeconomically deprived populations facing higher rates of exposure, incidence, and mortality (7, 23–25). These associations have been found to hold even at very local scales, highlighting the critical need to allocate more resources for pandemic recovery efforts on vulnerable populations as they are at higher risk of facing a syndemic rather than a pandemic (7, 26, 27). Additionally, studies have suggested that environmental factors such as air pollution and atmospheric conditions may play a significant role in the transmission of the virus (17, 28–31). “Indeed, exposure to air pollutants from both human-related emissions and natural events, such as particulate pollution and desert dust, can contribute to an increased diffusion of the virus” (17, 28–31).

Besides investigating the factors influencing the spread of SARS-CoV-2, research efforts have also been focused on understanding the patterns and intrinsic characteristics of the different waves of the pandemic. For instance, a study by Rader et al. (13) found that the peakedness of COVID-19 epidemics was influenced by population aggregation and heterogeneity. Specifically, the study found that epidemics in crowded cities were more spread over time and exhibited larger total attack rates compared to less populated cities.

Our study aims to analyze the various spatiotemporal factors influencing the spread of SARS-CoV-2 and examine how their impact may have evolved between March 2020 and April 2022. SARS-CoV-2 RT-PCR testing data georeferenced at a fine geographical scale over the canton of Vaud in Switzerland provide geolocated epidemiological time series data on COVID-19 within various geographical settings (i.e., municipalities and hectares). It offers an unpreceded opportunity to assess the influence of local factors in determining epidemic behaviors. Indeed, employing high spatial resolution data may provide insights into local variations that would be indiscernible at coarser geographic scales. The enhanced granularity could help inform local policies, targeting public health interventions where most needed and finely tuning them to fit the specific conditions and needs of the affected communities, thereby allowing for better resource allocation (6, 11, 32). We investigate an extensive range of sociodemographic and environmental factors that may influence the diffusion dynamics and geographical patterns of SARS-CoV-2 using advanced spatial and analytical methods. This geospatial approach also helps to address some of the shortcomings of previous studies such as the use of broader geographical scale and the use of models that do not consider spatial effects.

## Materials and methods

**Figure 1:**
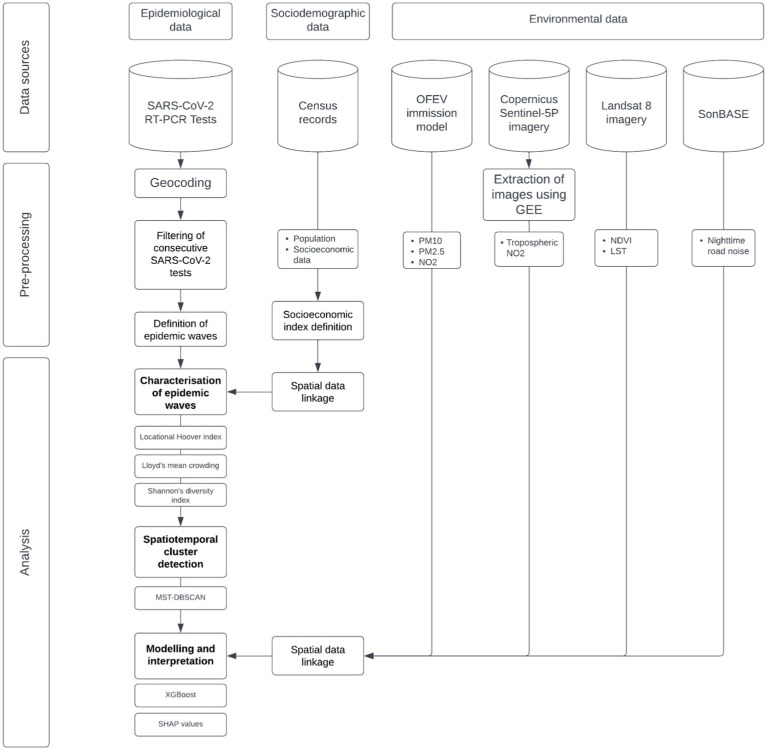
Flowchart of the study methodology. This chart outlines the approach used in our study, depicting the progression from data collection through to analysis. It highlights our data sources, including epidemiological, sociodemographic, and environmental datasets, and describes the sequential data processing operations. The methodology culminates in a modeling phase employing XGBoost, followed by an interpretative analysis using SHAP values to uncover the influence of the various factors on the diffusion dynamics and geographic patterns of SARS-CoV-2.

Figure 1 depicts the study’s methodological workflow, illustrating the data sources, pre-processing operations, and analyses.

### Data sources and preprocessing

COVID-19 testing and case data were obtained from the Institute of Microbiology (Lausanne University Hospital, CHUV). Data on socioeconomic factors, air pollution (PM10, NO_2_), noise pollution, vegetation (The normalized Difference Vegetation Index—NDVI), temperature (Land Surface Temperature—LST), population density at the hectare level, and population density around each hectare were collected from various sources, including census records, satellite imagery, and air and noise pollution immission models. All datasets were preprocessed to ensure compatibility.

#### Geocoding of the residential addresses

We geocoded the residential addresses of individuals who were tested for SARS-CoV-2 using an in-house offline procedure based on a Gestalt string matching algorithm. This algorithm was chosen for its robustness in handling a variety of misspellings and inconsistencies in address formats (33). This algorithm matches each residential address against a comprehensive dataset of all existing addresses in the canton of Vaud. The match exhibiting the highest level of similarity was then retained if the similarity was above 80% (*n* = 241,775, 85.4%). An 80% similarity threshold was set based on preliminary analyses that demonstrated a balance between match accuracy and inclusion of valid addresses. In total, 41,360 tests were not geocoded for various reasons. First, individuals residing outside of the study area, namely beyond the canton of Vaud or in other countries, were not included in the analysis (*n* = 31,506, 11.1%). This exclusion does not impact our analysis, as these tests fall outside our study scope. Second, a further group whose residential address could not be geocoded was also omitted (*n* = 9,854, 3.5%). Finally, in instances where the street number was missing, the addresses were geolocated at the centroid of the street (*n* = 5,918, 2.4%).

#### Filtering of consecutive SARS-CoV-2 tests

To accurately assess the incidence of SARS-CoV-2 infection in the study population, we filtered out consecutive RT-PCR tests performed within 20 days (*n* = 47,093, 19.5%). This prevented repeated testing of recent positive cases and emphasized unique infections. This approach helped ensuring that the dataset accurately represented distinct SARS-CoV-2 infections throughout the study. The final dataset comprised a total of 194,682 tests.

#### Defining epidemic periods

The SARS-CoV-2 pandemic has undergone multiple waves and mutations of the virus, affecting transmission rates and testing outcomes. We divided our dataset into five periods representative of the five major epidemic waves to analyze their impacts: the initial outbreak of the pandemic in early 2020 (Period 1, Feb 3, 2020–June 30, 2020), the second wave that occurred later that year (Period 2, July 1, 2020–Dec 15, 2020), the third wave in early 2021 (Period 3, Dec 16, 2020–May 7, 2021), the arrival of the severe Delta variant (Period 4, May 8, 2021–Nov 28, 2021), and the highly transmissible Omicron variant emergence (Period 5, Nov 29, 2021–April 15, 2022). This division allowed us to evaluate the characteristics of each wave and the potential evolution of the determinants of diffusion.

#### SARS-CoV-2 RT-PCR testing data

Our analyses included 41,672 positive SARS-CoV-2 RT-PCR tests from a total of 283,135 tests administered to 138,774 residents of the canton of Vaud (population 800,000), Switzerland, between March 2, 2020, and April 15, 2022. The testing procedure relied only on quantitative real-time PCRs and has been described in detail in previous studies (8, 10). The study received approval from the Cantonal Research Ethics Commission of Vaud (CER-VD), Switzerland (n°2020-01302).

#### Sociodemographic data

Demographic data used in this study were obtained from the Swiss Federal Population and Household statistics (34), which provides detailed information on the population at the hectometric scale. This data include population counts and demographic characteristics. This data were used to provide an accurate picture of the population distribution in the study area.

We calculated a socioeconomic deprivation index at the hectare level, using socioeconomic data at the hectometric scale^1^ and a methodology developed by Lalloué et al. (35), which has been previously used in studies investigating socioeconomic disparities in health (7, 36). This methodology involves a series of principal component analyses to identify and remove redundant variables, select key variables of interest, and combine them into a single index that reflects socioeconomic deprivation (35). The socioeconomic index was normalized to a scale ranging from 0 to 1, where a value of 0 represents the highest level of socioeconomic deprivation, and a value of 1 denotes the lowest level of deprivation. This standardization facilitates a more intuitive interpretation of the index, aligning higher values with less deprivation.

#### Environmental data

Six environmental variables that represent the living environment of the population were considered: nighttime road noise, a vegetation index (NDVI), an estimate of ground surface temperature (LST) and air pollution markers (NO_2_, PM10, PM2.5). These factors help identify areas with conditions potentially promoting transmission. Nighttime road noise data were produced by the Swiss Federal Office for the Environment (OFEV) and compiled in the SonBASE database (37), served as a proxy for urban density and road traffic activity. Nighttime noise was selected as it represents the longest exposure at the residential address. This database provides a value in dB(A) for the whole territory with a resolution of 10 m. From these values, we calculated the average nighttime car noise value for each populated hectare of the Vaud territory.

The normalized difference vegetation index (NDVI) and land surface temperature (LST) were derived from Landsat 8 satellite images of the Lake Geneva region, taken during the summer of 2021 (20.07.2021) (38). The NDVI is a satellite-derived measure indicating the presence and condition of vegetation, with higher values signifying healthier vegetation while LST measures the heat radiated by land surfaces, also derived from satellite data, informing studies on urban heat islands. Both these indices serve as critical environmental variables to quantify local variations in temperature, humidity, and urbanicity levels (39, 40).

Air pollution data were obtained from two sources. First, 2020 Meteotest’s immission model commissioned by the OFEV (41) provided information about air pollution levels (NO_2_, PM10, PM2.5) at a 20-m resolution. Despite being anterior the COVID-19 pandemic, the immission model provide valuable information on the baseline conditions and long-term exposure to air pollutants in the study area. Second, to account for short-term exposure air pollution, daily nitrogen dioxide (NO_2_) levels were obtained from satellite imagery via Google Earth Engine (42). To obtain daily average tropospheric NO_2_ concentrations, we extracted and processed Sentinel-5 Precursor imagery (3.5 × 7 km^2^ spatial resolution) using algorithms adapted from Ghasempour et al. (43). We aggregated the daily average concentrations by month resulting in a time-series of monthly tropospheric NO_2_ concentrations with comprehensive coverage of the study area during the study period.

### Characterization of the epidemic waves

Three indices were calculated for each epidemic period and municipality of the canton of Vaud: the Inverse Shannon entropy index to evaluate the temporal clustering of cases, Lloyd’s index of mean crowding to understand population structure, and the Hoover index to compare the spatial distribution of the population to the spatial distribution of COVID-19 cases.

#### Inverse Shannon entropy

To evaluate how temporally clustered COVID-19 cases are within each municipality, we used the Shannon diversity index. For a specific municipality, we established the incidence distribution as the ratio of COVID-19 cases *j* taking place on day *i*. The Shannon index, represented by (1) is based on the disease incidence curve for each location, making it less susceptible to variations in reporting rates across municipalities.


(1) $$-{\left(\sum \left(p_{ij}*\log\left(p_{ij}\right)\right)\right)}^{-1}$$

The index achieves its highest value when all cases occur on 1 day and its lowest value when the epidemic has an equal number of cases on each day.

#### Locational Hoover index

The Hoover index is a widely used measure to assess trends of concentration in the distribution of a population. To evaluate the progressive spread of COVID-19 cases, we used the locational Hoover index, which measures spatial imbalance between two variables in a given geographic area (44). It compares the proportion of the municipality’s total population residing in a particular hectare to the proportion of COVID-19 cases occurring in that same hectare during a specific time period. This provides a way to understand whether COVID-19 cases are clustered in certain areas or distributed more evenly throughout the municipality. Values closer to 100 indicate concentration in few hectares, while those close to zero suggest a more homogeneous spreading (44). In cases where a hectare intersected with multiple municipalities, it was assigned to the municipality having the larger population.

#### Lloyd’s mean crowding

To better understand differences in population structure across municipalities, we employed the Lloyd’s index of mean crowding (45), considering each hectare’s population count within each municipality. Higher values of Lloyd’s index indicate a more spatially clustered population structure while lower values indicate a population structure that is more evenly distributed.

### Spatiotemporal cluster detection

To monitor and analyze the spatiotemporal patterns of SARS-CoV-2 diffusion, we used the MST-DBSCAN (modified space–time density-based spatial clustering with application with noise) algorithm (46). This method, a modified version of the well-established DBSCAN algorithm, identifies clusters of arbitrary shapes and is adept at capturing complex patterns irrespective of administrative boundaries (47). The settings we used included a spatial distance of 200 m, a minimum period value of 1 day, and a maximum period value of 14 days.

Utilizing the MST-DBSCAN algorithm, we investigated the spatial and temporal variations in the dynamics of COVID-19 waves. This allowed us to identify and monitor spatiotemporal clusters throughout the study period based on spatial and temporal proximity (45, 47). Importantly, it enabled us to monitor cluster persistence.

### Cluster persistence

Cluster persistence, defined as the duration from the emergence to the disappearance of a cluster, was analyzed to understand diffusion dynamics and pinpoint areas with prolonged persistence (7). While clusters identified through MST-DBSCAN can take arbitrary shapes, we projected them onto the populated hectares in the canton of Vaud to capture the duration each hectare remained within a cluster. Hectares experiencing multiple cluster episodes (i.e., repeated emergence and disappearance) were assigned the cumulative duration spent within a cluster.

### Modeling

#### eXtreme gradient boosting

To evaluate the associations between cluster persistence and sociodemographic and environmental features, we employed the eXtreme Gradient Boosting (XGBoost), a widely popular machine learning algorithm that has been used in many supervised classification and regression applications (48–50), including for COVID-19 research (51, 52). XGBoost is a gradient boosting algorithm that iteratively ensembles decision trees using gradient descent algorithm to minimize model error (53).

We assessed multicollinearity using the Variance Inflation Factor (VIF), considering values above 10 to indicate high multicollinearity. To mitigate issues of multicollinearity, we combined Land Surface Temperature, NDVI, and Nighttime car noise into an “Urban type index” using principal component analysis. Similarly, an “Air pollution index” was derived from the three measures of air pollution provided by the immission model: NO2, PM10, and PM2.5. To further prevent multicollinearity, the air pollution and the socioeconomic deprivation indices were evaluated in separate models.

Given the significant spatial autocorrelation in the distribution of cluster persistence (Supplementary Figure S1, Moran’s I = 0.95, *p* < 0.001), we incorporated geographic coordinates of each hectare’s centroid into the multivariable models to capture these spatial dependencies. XGBoost models that include geographic coordinates have been shown to adequately capture spatial effects (i.e., spatial autocorrelation and spatial heterogeneity) when compared to classical statistical spatial modeling methods such as the spatial lag model and the Multiscale Geographically Weighted Regression (MGWR); these spatial effects being captured through the coordinates themselves, their interaction (longitude * latitude) and the interaction between coordinates and non-spatial features (54).

In addition, machine-learning approaches like XGBoost generally require fewer assumptions about the underlying processes and perform well at identifying patterns in large datasets with complex nonlinear interactions (55). In comparison, model selection in spatial modeling can be computationally challenging, particularly due to the need for additional calculations such as fitting local regression at each location (54).

One limitation of XGBoost is that it can be difficult to interpret the importance of individual features in the model. To improve interpretability, we used SHapley Additive exPlanations (SHAP), an effective interpretability technique for machine learning models (56). SHAP is a game-theoretic approach that assigns to each model feature a numerical value that represents its contribution to the final prediction. This allows for a transparent understanding of how the model provides predictions. This is particularly important for epidemiology and public health applications where interpretability is critical. However, it should be noted that SHAP values, unlike coefficients in a regression model, represent partial dependence. They characterize the contribution of a specific feature to the difference between the actual prediction and the mean prediction while accounting for other factors in the model (56). This distinction is crucial, as SHAP values provide a more nuanced understanding of the relationships between variables in our XGBoost models by considering complex interactions that may not be captured by traditional regression coefficients (57).

Anticipating the potential for reporting bias—due to variations in testing rates possibly leading to more reported cases—we adjusted for differences in testing rates across areas and time periods to account for these disparities across different areas and time frames. We determined the testing rate for each hectare by dividing the number of tests by the population and calculated rates for each time period to account for changing testing practices.

To estimate the XGBoost model’s performance, the dataset was split into an 80% training and 20% testing partition. The model was trained on the training set, and its generalization capacity and predictive accuracy were assessed using the coefficient of determination (*R*^2^) and root mean square error (RMSE) on the testing set. The hyperparameter optimization procedure is described in the Supplementary material Section S1: “Hyperparameter Optimization.”

Following the primary analysis with XGBoost, we conducted a sensitivity analysis to further investigate the seemingly low influence of the SES index on cluster persistence.

#### Sensitivity analyses

Given the initial results suggesting a weak association between the SES index and cluster persistence, we sought to assess the robustness of our findings by replicating the methodologies from previous work (7), which demonstrated a significant association between socioeconomic status and cluster persistence. Consequently, we used a Cox Proportional Hazards (PH) model, adjusting for population density and testing rates to control for confounding.

## Results

### Description of the temporal and spatial clustering of COVID-19 cases

**Figure 2:**
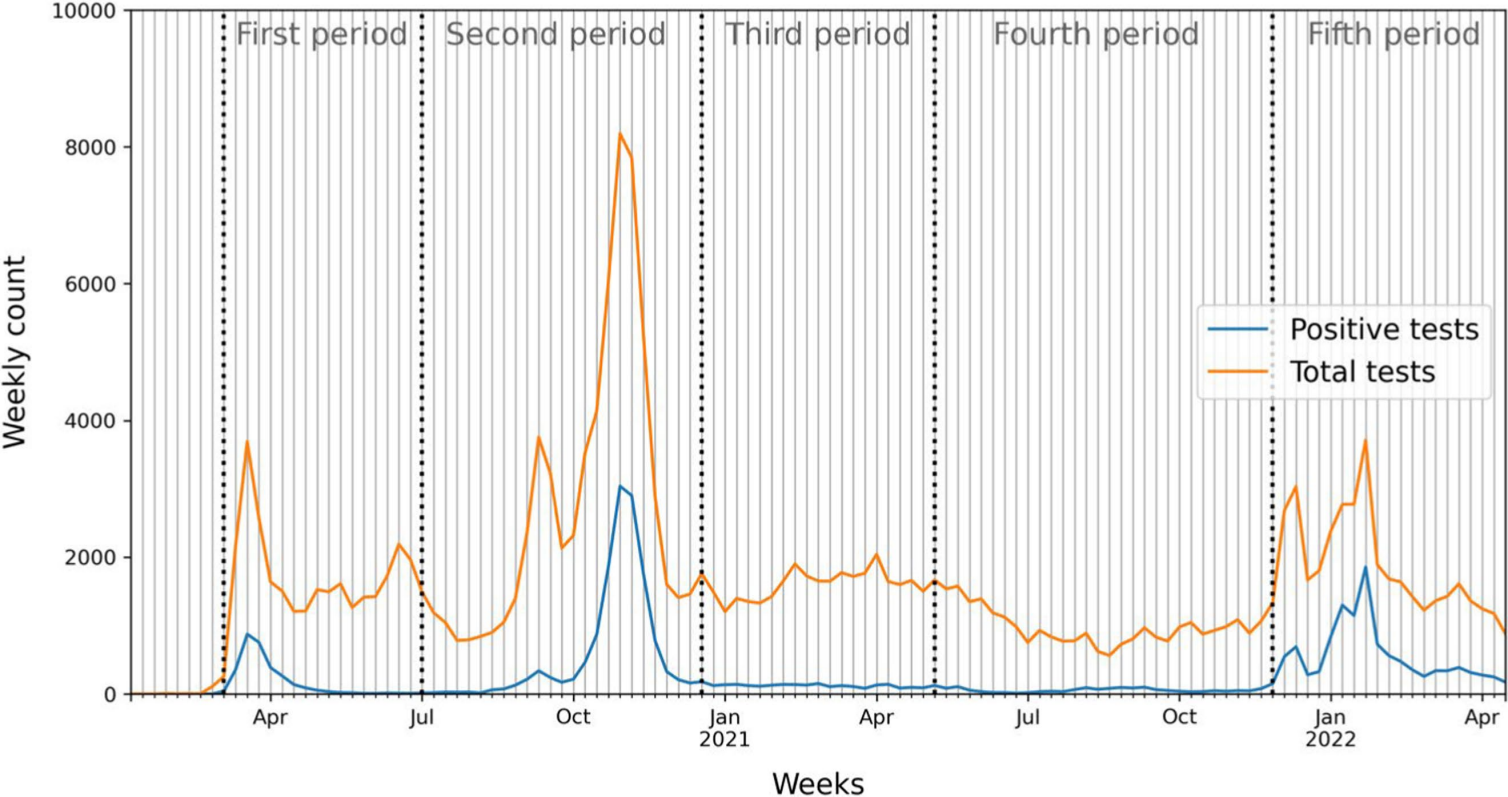
Weekly distribution of total SARS-CoV-2 RT-PCR tests and positive tests throughout the study period. Dotted lines correspond to the five defined periods.

The time-series of SARS-CoV-2 RT-PCR testing data allows to track the weekly count of tests and positive cases across the study’s five defined periods (Figure 2). Three distinct peaks emerged, corresponding to the main pandemic waves that have been documented in the Canton de Vaud. The first wave was observed during the onset of the pandemic, followed by a second wave in the last months of 2020 and a third peak linked to the Omicron variant in late 2021 and early 2022 (fifth period).

The second peak displayed the highest number of positive cases and volume of tests, indicative of a substantial surge in virus prevalence and testing capacity. The third period was characterized by a low positivity rate with moderate testing intensity (Supplementary Figure S2). The fourth period shows a decrease in both positive cases and test number. During the fifth period, the Omicron-associated peak underscored the emergence of this highly transmissible variant with a high positive rate of around ~50% (Figure 2; Supplementary Figure S2) reached around the end of January 2022.

**Figure 3:**
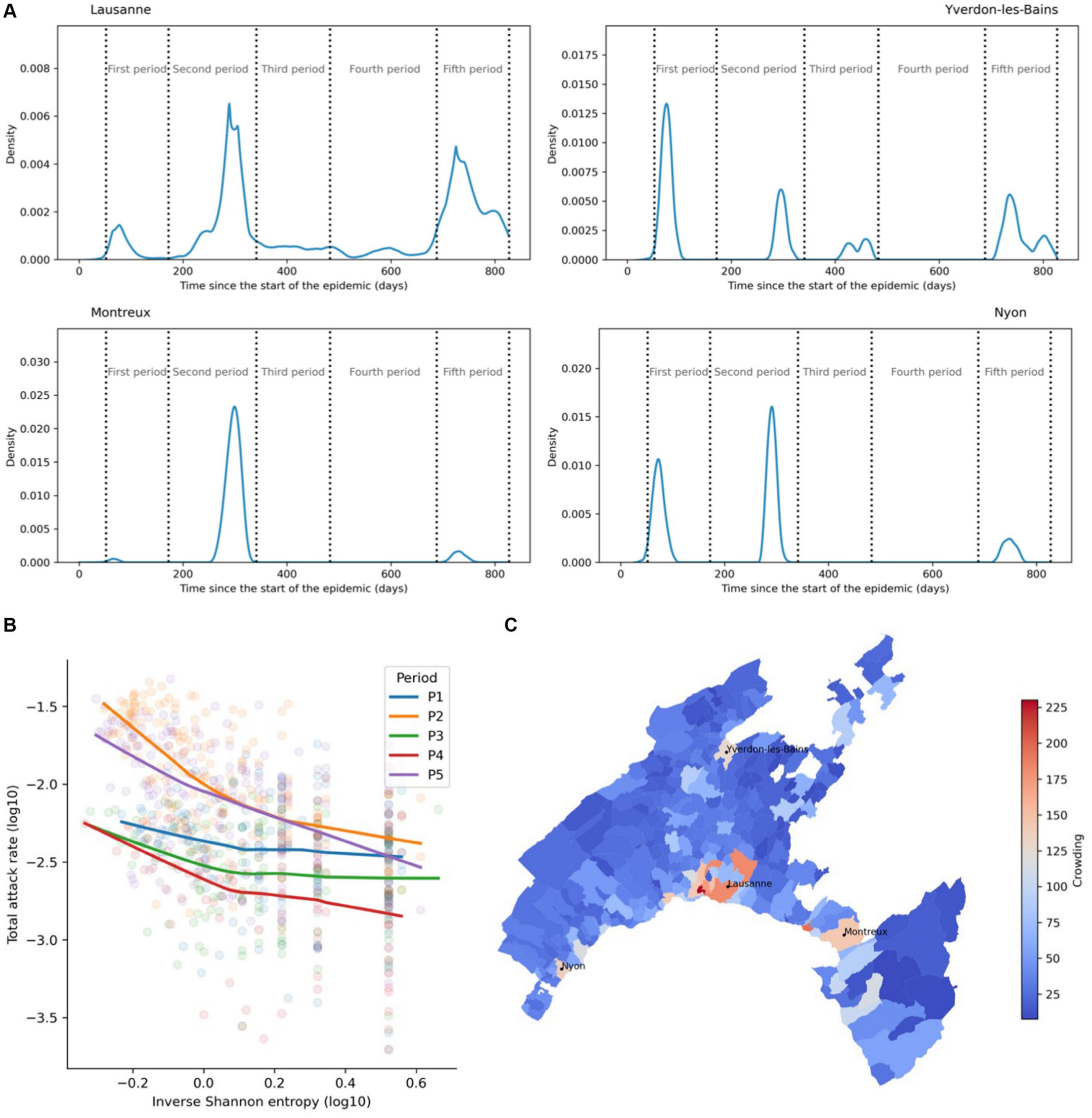
Epidemic curves, temporal clustering, and population structure. **(A)** Examples of epidemic curves across the study period for four municipalities of the canton and showing the percentage of the total cases happening on any given day. Lausanne and Yverdon-les-Bains (YLB) have less peaked epidemics than Montreux and Nyon. **(B)** Relationship between the Shannon index and the final attack rate for municipalities of the canton of Vaud and each period (p1 = first period, ..., p5 = fifth period). Lines correspond to LOWESS curves fitted for each period. **(C)** Lloyd’s index of mean crowding for each municipality of the canton of Vaud.

The epidemic curves reveal distinct epidemic shapes across different geographical and temporal contexts, as shown when specifically looking at four major towns (Lausanne, Yverdon-les-Bains, Montreux, and Nyon) and five epidemics waves (Figure 3A). The most populated area of Vaud canton, the town of Lausanne (population ~ 140,000) exhibited the least peaked epidemics while the distribution of cases over time corresponds to the one of the cantons. The three other municipalities showed higher peakedness but distinct epidemic behaviors. Yverdon-les-Bains (YLB, pop. ~ 30,000) had a very high peak of cases during the first period and relatively low peak during the second period. In Montreux (pop. ~ 26,000), cases were mostly concentrated in the second period with only a little fraction distributed in the first and fifth period while in Nyon (pop. ~ 22,500) cases were mostly distributed among the two first periods with a very low fraction present in the fifth. These two smaller cities also have in common the almost complete absence of cases during the fourth period.

Descriptive statistics provided more information on the different transmission dynamics. The Inverse Shannon entropy index values for the first, and third periods were almost equal at 0.24, and 0.22, respectively (Table 1). The fourth period showed the highest peakedness (0.29) while the second and fifth periods showed the lowest values at 0.14 and 0.16, respectively (Table 1). The total attack rate in each municipality was negatively correlated with the inverse Shannon entropy index in each period suggesting that flatter epidemics (i.e., less peaked) have a higher total attack rate (Figure 3B). We observed shared patterns between periods 1, 3, and 4; exhibiting a flatter profile and between periods 2 and 5 that have a steeper slope. The LOWESS curves suggest a negative relationship that tends to attenuate at Shannon entropy index values (log-transformed) above 0 (Figure 3B). Lloyd’s index of mean crowding provided valuable insights into the spatial structure of the population in each municipality taking into account both population density and how density is distributed. For instance, Montreux has a relatively lower population density compared to Nyon and YLB but exhibits a higher crowding value due to the patchiness of the distribution of its population (Figure 3C; Supplementary Figure S3).

**Table 1:** Characteristics of the five periods.

Period	Overall	P1(First wave)	P2 (Second wave)	P3 (Third wave)	P4 (Delta)	P5 (Omicron)	*p*-value
Number of clusters	3,175	280	1,482	121	47	1,228	
Peakedness, median [Q1, Q3]	0.20 [0.09, 0.39]	0.24 [0.13, 0.39]	0.14 [0.08, 0.29]	0.22 [0.12, 0.40]	0.29 [0.13, 0.42]	0.16 [0.07, 0.31]	<0.001
Hoover index, median [Q1, Q3]	79.8 [0.0, 92.5]	86.5 [69.4, 94.5]	75.1 [50.1, 87.9]	81.8 [0.0, 93.8]	0 [0.0, 92.9]	78.0 [55.0, 91.4]	<0.001
Cluster persistence (days), median [Q1, Q3]	4 [2, 9]	6 [3, 10]	4 [2, 9]	5 [2, 9]	5 [2, 10]	4 [2, 9]	0.004
Number of positive tests, median [Q1, Q3]	1 [1, 3]	2 [1, 3]	2 [1, 4]	1 [1, 2]	1 [1, 3]	1 [1, 3]	0.518

Descriptive statistics, including number of clusters, peakedness, hoover index, cluster persistence, and number of positive tests of the pandemic overall and over the five different periods.

**Figure 4:**
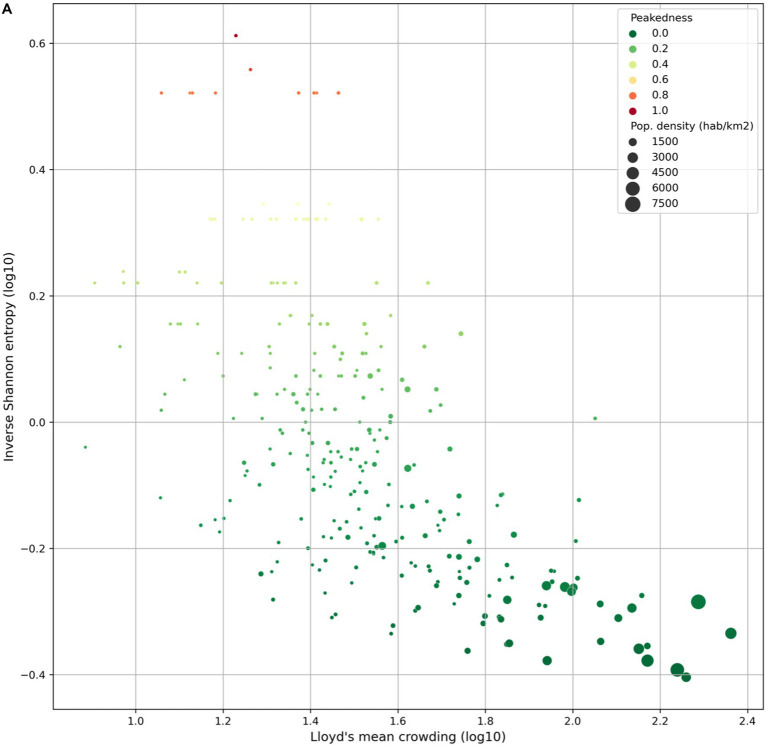

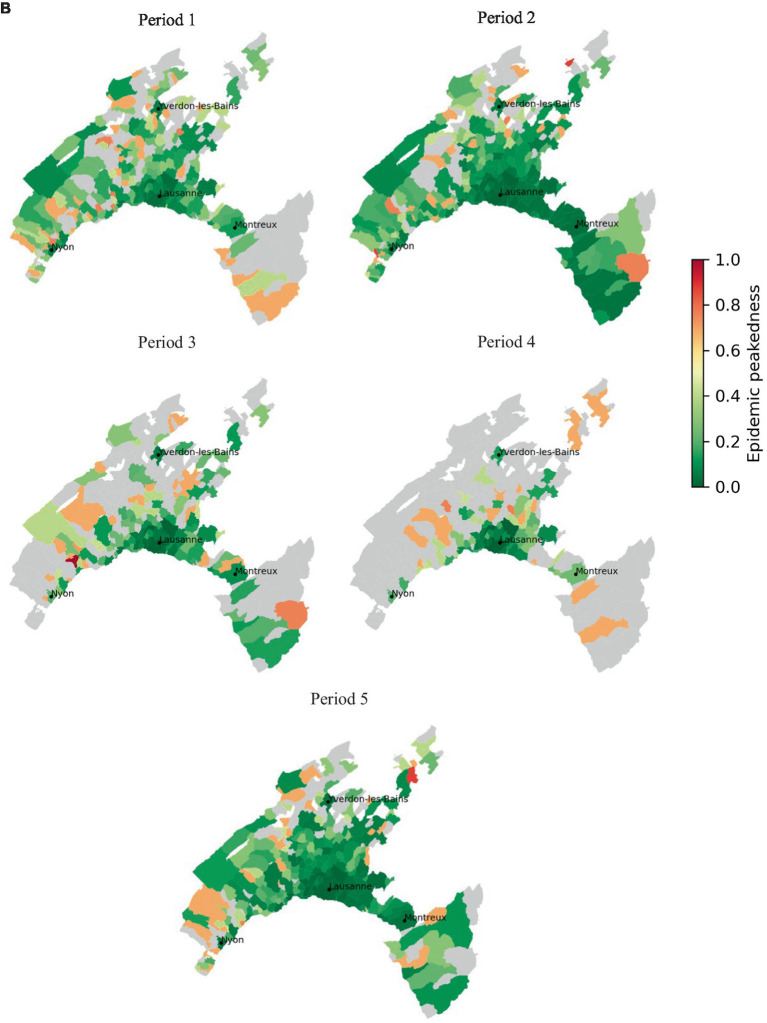
Population crowding and epidemic peakedness. **(A)** Relationship between the Shannon index and the Lloyd’s index of mean crowding. **(B)** Maps of the Shannon index (scaled from 0 to 1) for each defined period of the study. Municipalities with no reported cases during a period are shown in gray.

Our analysis showed a strong correlation between population structure and the peakedness of the pandemic waves (Figure 4A). In densely populated urban areas, the crowding index was significantly higher and the peakedness lower compared to sparsely populated municipalities. The spatial distribution of the temporal clustering of cases for each period (Figure 4B) illustrates the wide variations of the Shannon index (scaled from 0 to 1) across different municipalities and periods.

**Figure 5:**
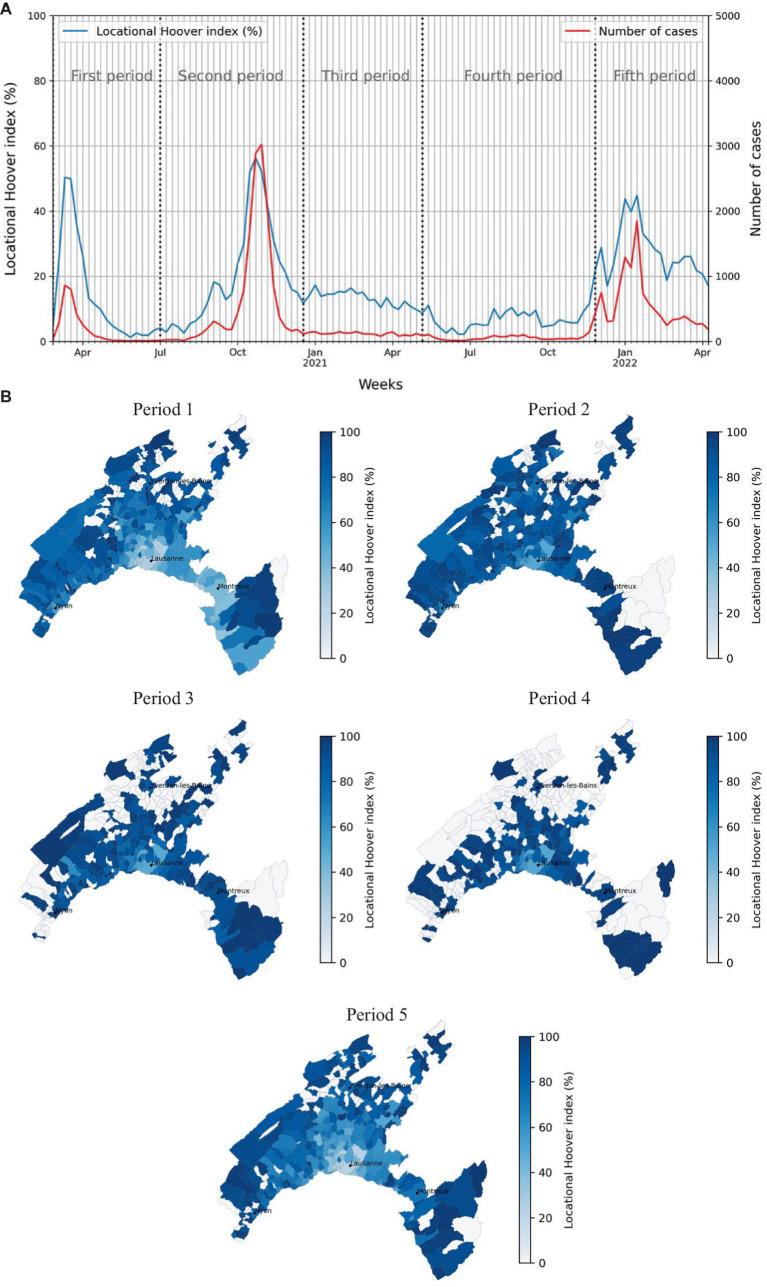
Locational Hoover index. **(A)** Weekly locational Hoover index over the study period. **(B)** Maps of the locational Hoover index for each defined period of the study. Values closer to 100 indicate concentration of SARS-CoV-2 cases in few hectares of the municipality, while those close to zero suggest a more homogeneous spreading of cases in the municipality.

The time-series analysis of the weekly locational Hoover index (%) and total positive cases, revealed that despite substantial differences in the number of weekly positive cases at the peak of periods 1 and 2, the locational Hoover index values for these two periods were strikingly similar (Figure 5A). This apparent paradox is likely due to different testing strategies, due to higher tests capacity during the second period. The locational Hoover index calculated for each period had median values of 86.5, 75.1, 81.8, 0, and 78.0 for the first, second, third, fourth, and fifth periods, respectively (Table 1). These findings suggest that the second period had the most homogeneous distribution of cases while the fourth period had the most unequal (i.e., spatially clustered) distribution of cases within the population. To gain more insight on it, we mapped the locational Hoover index across different periods, allowing for an easy comparison of the spatial patterns of case concentration (Figure 5B).

### Spatiotemporal cluster detection and cluster persistence

**Figure 6:**
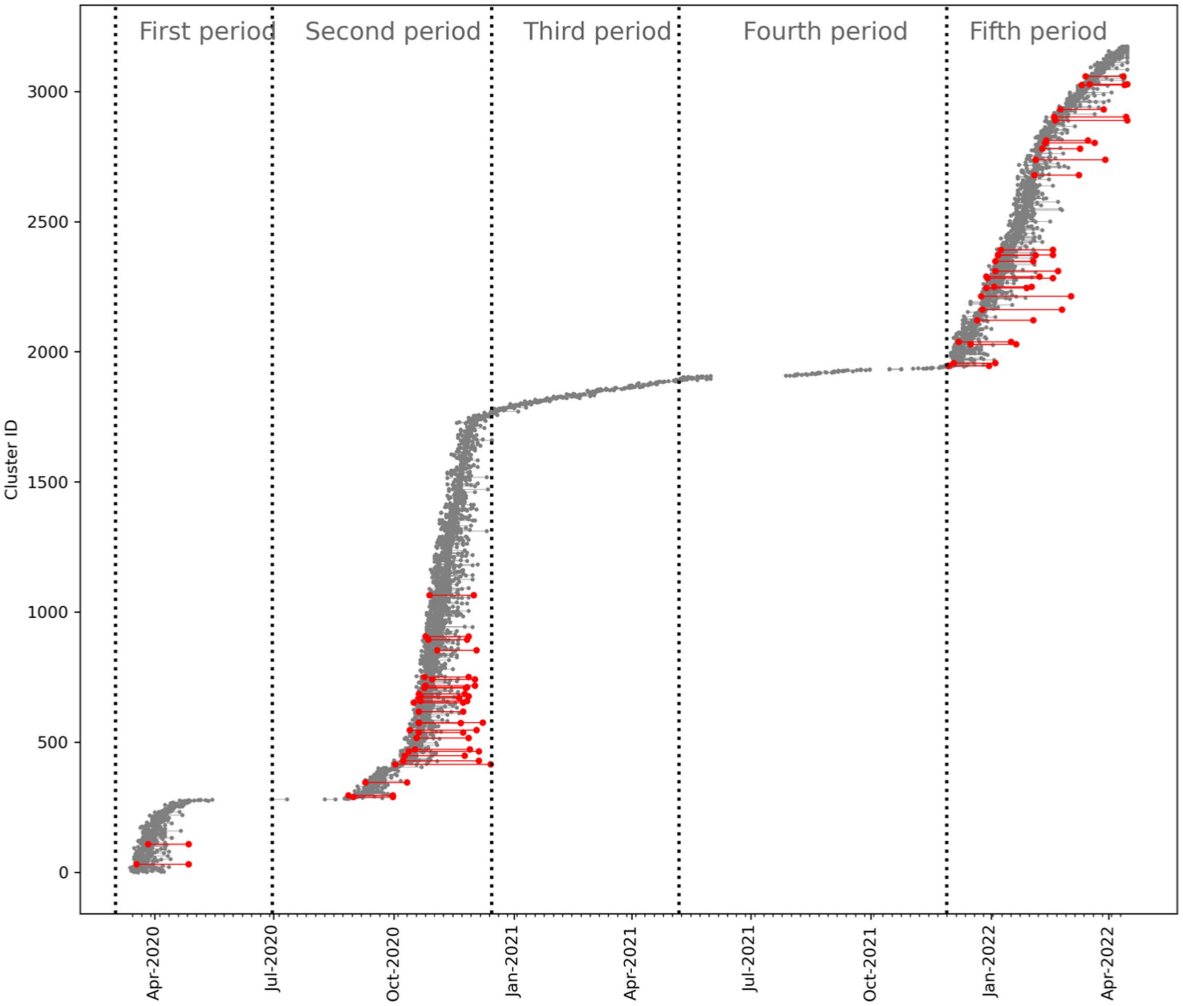
MST-DBSCAN clusters. Timeline of MST-DBSCAN clusters identified throughout the study period. Each cluster is indicated as a line going from the date of its emergence to the date of its disappearance. Clusters that persisted for more than 30 days are highlighted in red.

The MST-DBSCAN analysis identified a total of 3,175 clusters with periods 2 and 5 exhibiting the highest number of clusters (Table 1). Figure 6 illustrates the emergence and disappearance of these clusters throughout the study period. Among the 3,175 clusters, 3,158 emerged and disappeared within the same period, while 17 overlapped between two periods (P1-P2: 1, P2-P3: 7, P3-P4: 3, and P4-P5: 6). The differences in median cluster persistence across periods were statistically significant (Table 1). Cluster persistence was positively associated with the number of positive RT-PCR tests from cluster emergence to disappearance (Pearson’s *r* = 0.36, 95% confidence interval (CI), 0.33 to 0.36, *p* < 0.01) (Supplementary Figure S4). Additionally, the number of clusters persisting for more than 30 days varied considerably among the periods. Only two were present in period 1, while 28 emerged in period 2, none in periods 3 and 4, and 27 appeared in period five (Figure 6).

The spatial distribution of cluster persistence shown on the map reveals a pronounced concentration of longer-lasting clusters in urban areas, particularly around the city of Lausanne (Supplementary Figure S1). This pattern underscores the potential influence of higher population density and urban activity on the sustained transmission of SARS-CoV-2, factors that were considered in the subsequent modeling analyses.

### Determinants of cluster persistence

#### Univariate analyses

We first conducted univariate XGBoost model analyses for each demographic, socioeconomic, and environmental feature independently and for the whole study period. This step involved fitting separate XGBoost models for each individual feature, which allowed us to explore the potential relationship between each feature and cluster persistence in isolation. This initial univariate analysis served as a preliminary assessment of the relevance and potential importance of each feature in predicting the outcome of interest. The following features were evaluated: population density (*Population*) in the hectare, population density in the surroundings (*Lagged population 200 m, Lagged population 8-NN, Lagged population 24-NN*), testing rate [*Testing rate (%)*], socioeconomic deprivation index (*SES index*), vegetation index (*NDVI*), the land surface temperature (*LST*), the nitrogen dioxide concentration (*NO_2_*), the 10 and 2.5 microns or less particulate matter concentration (*PM10 and PM2.5*) extracted from the immission models (see section Data sources and preprocessing), the tropospheric NO_2_ concentration average for each period [*Tropospheric NO_2_ (periodic avg)*], the nighttime car noise (*Nighttime car noise*), and the longitude (*E*) and latitude (*N*). The lagged population 24-NN corresponds to the average population in the 24 nearest populated hectares. This number was chosen to match the radius of 200 m used in the MST-DBSCAN analysis used for spatiotemporal cluster detection and thus the cluster persistence definition.

While the three predictors capturing population density in the surroundings demonstrated strong performance, the “Lagged population (24-NN)” was the most promising predictor and was retained for the multivariable models. The “Testing rate (%)” presented a moderately high *F*-score of 1,402 and an *R*^2^ value of 0.34, indicating the importance of adjusting for it in subsequent analyses. Air pollution features such as NO_2_, tropospheric NO_2_, PM2.5 and PM10 displayed reasonable *F*-scores, *R*^2^ and, RMSE values, pointing to their usefulness in predicting cluster persistence (Table 2).

**Table 2:** Overall model accuracy of the univariate XGBoost models.

Feature	*F*-score	*R* ^2^	RMSE
Population	814	0.33	34
Lagged population (8-NN)	1,492	0.58	26.8
Lagged population (24-NN)	1,666	0.66	23.6
Lagged population (200 m)	1,709	0.57	27
Testing rate (%)	1,402	0.34	33.6
SES index	1,900	−0.03	42
NDVI	1,736	0.02	40.8
LST	1,830	0.03	40.9
NO_2_	2,198	0.24	36.1
PM10	2,144	0.29	34.8
PM2.5	1,828	0.36	33.2
Tropospheric NO_2_	1,796	0.2	37.1
Nighttime car noise	1,995	0.006	41.3
E	1,374	0.28	35.2
N	1,243	0.28	35

For each analyzed features, model’s accuracy (*F*-score), the proportion of variance in the dependent variable that can be explained by the independent variable (*R*^2^) and the root of the Mean Square Error (RMSE) are computed.

However, some features like SES index, NDVI, Nighttime car noise showed low *R*^2^ values and limited predictive power. Interestingly, SES index has a high *F*-score of 1,900 but a negative *R*^2^ value, suggesting that it may not contribute meaningfully to the model’s explanatory power (Table 2). The sensitivity analyses evaluating the relationship between the SES index and cluster persistence revealed a significant association between the SES index and cluster persistence [Hazard Ratio (HR) = 0.49, *p* < 0.005], but a low Concordance index (C-index = 0.54), which is only slightly better than a random guess (0.5), indicating limited predictive accuracy.

Finally, we complemented these results with multivariable models to account for potential interactions and combined effects of multiple features.

#### Multivariable analyses

We estimated the joint effect of all spatial and non-spatial features on cluster persistence by fitting separate multivariable XGBoost models corresponding to each period.

Supplementary Figure S5 shows the SHAP summary plots for the 16 top contributing features at each of the five periods. The lagged population was a key feature, highlighting the importance of adjusting for surrounding population density in our models. The location effects were also essential in the model, as illustrated by the range of SHAP values of the E and N geographic coordinates (Supplementary Figure S5). The contribution of the location effect on cluster persistence, measured by SHAP values of E and N is shown in Supplementary Figure S6. These display a clear spatial pattern with locations in red representing hectares contributing positively, while those in blue depict hectares with a negative impact. It is crucial to emphasize that these effects account for all other features in the model, highlighting the unique contribution to cluster persistence stemming only from the location effect. The areas with positive contribution on cluster persistence are mainly located in the urban area of Lausanne and to its East side.

Air pollution—captured by the air pollution index and tropospheric NO2—was an important feature in all periods (Supplementary Figure S5). In period 1, 3, and 5, the air pollution index was the most contributing features after the lagged population density. In period 2, the air pollution index was most important feature of the model. In period 4, tropospheric NO_2_ had a great contribution to the model. The other features, the urban type and SES indices, and the testing rate, contributed only very slightly to the models. There were several interactions between spatial and non-spatial features but of relatively low contribution to the models. Regarding overall fit, *R*^2^ and RMSE values are summarized in Supplementary Table S1.

**Figure 7:**
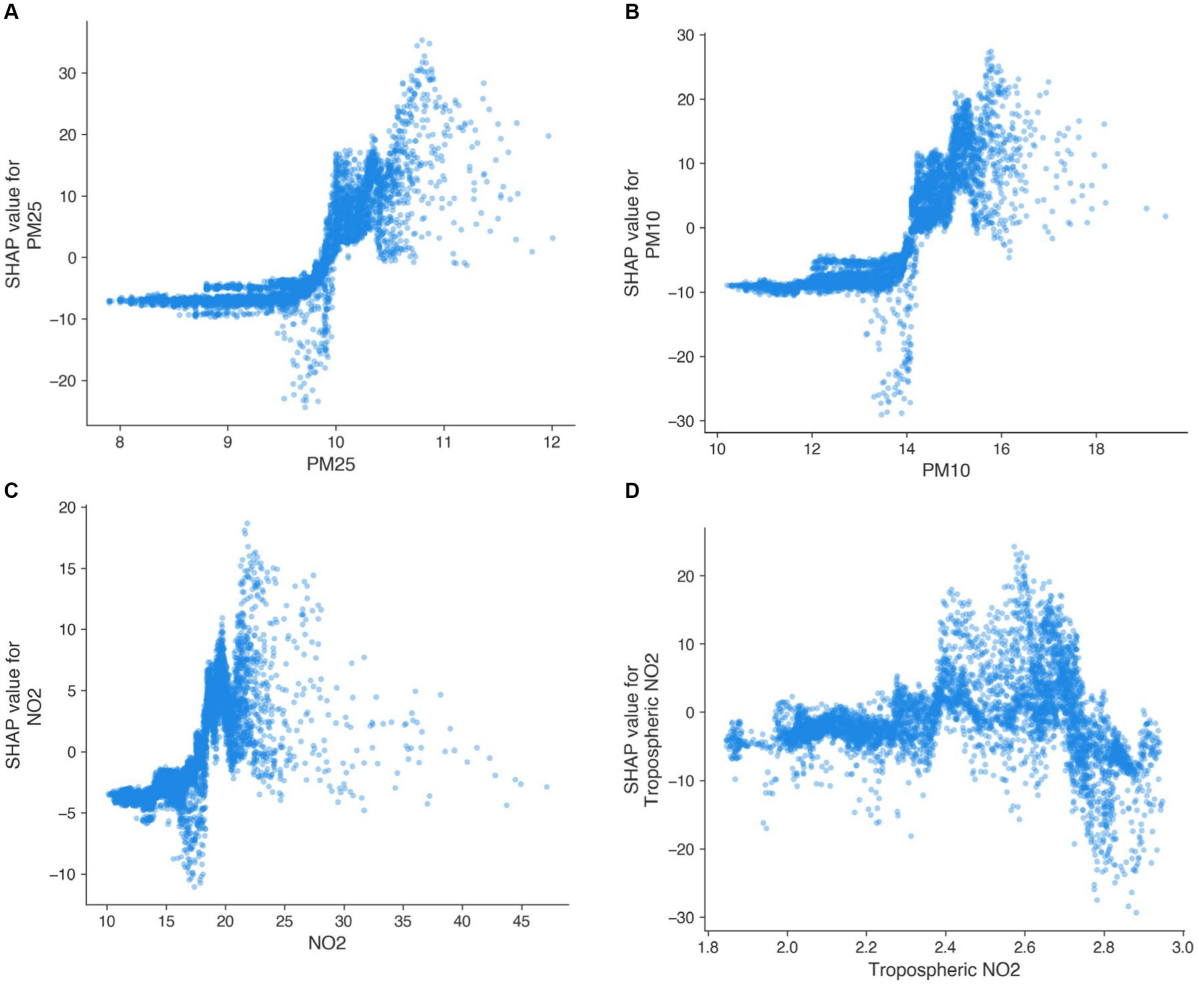
SHAP dependence plots showing the relationship between air pollutants and cluster persistence. **(A)** PM2.5, **(B)** PM10, **(C)** NO_2_, and **(D)** Tropospheric NO_2_.

In addition to the comprehensive XGBoost model incorporating all features, we also fitted separate models that focused on each air pollutant and on the SES index individually, along with location effects (E and N) and adjustments for population density [“Population” and “Lagged population (24-NN)”; Figure 7]. These analyses were conducted for the whole study period and specifically within the Lausanne urban area to ensure a purely urban context, avoiding potential residual confounding effects arising from urban versus rural comparisons. By conducting these separate analyses, we isolated the potential impact of each air pollutant on cluster persistence and examined their relationships with location and population density.

Across all air pollutants derived from the immission model, there was a clear pattern where higher concentrations were generally associated with higher cluster persistence. However, their relationships are non-linear and present a threshold at around 10.0 μg/m^3^ for PM2.5 (Figure 7A), 14.0 μg/m^3^ for PM10 (Figure 7B), and 16.0 μg/m^3^ for NO_2_ (Figure 7C). For PM10 and NO_2_, these thresholds are below the annual average immission limit values defined by the Swiss Air Pollution Control Ordinance (i.e., 20 μg/m^3^ for PM10 and 30 μg/m^3^ for NO_2_) (41). For PM2.5, the relationship’s threshold was right at the annual average immission limit value of 10.0 μg/m^3^. The relationship between tropospheric NO_2_ was less clear with a slight positive relationship until 2.7 mol/m^2^ followed by a negative relationship (Figure 7D), potentially due to the coarser spatial resolution.

In terms of variable importance, the analysis showed patterns similar to the multivariable models fitted for the whole study area and by period, with air pollutants showing a high contribution to the model and the SES and urban type indices showing a relatively modest contribution (Supplementary Figure S7).

## Discussion

### Summary of main findings

Our study examines the demographic, socioeconomic, and environmental determinants of SARS-CoV-2 diffusion and spatiotemporal dynamics at high spatial resolution. Most existing studies examine demographic (e.g., density, human mobility), socioeconomic, or environmental factors in isolation. Our work advances a more holistic approach by combining these variables with precisely geolocated SARS-CoV-2 testing data and advanced modeling techniques. Our main findings reveal a positive non-linear relationship between air pollution and cluster persistence with thresholds equal or below the annual average immission limit values for PM2.5, PM10, and NO_2_. Additionally, we identified stable diffusion characteristics across periods and no significant contribution of the socioeconomic index to cluster persistence.

### Comparison with existing literature

Our analysis using epidemic peakedness, Hoover index, and Lloyd’s mean crowding reveals SARS-CoV-2’s spatiotemporal diffusion dynamics, providing insights into case spatiotemporal distribution, population structure, and degree of clustering. While our findings on peakedness aligns with previous work conducted on influenza at the city-scale (58) and SARS-CoV-2 at the prefectural level in China (13), we were able to identify these patterns at much higher spatial resolution.

There is an ongoing debate about the association between air pollution, SARS-CoV-2 infection, and COVID-19 severity. While the study of this relationship is complex (59), several potential biological mechanisms underpinning these associations have been identified, ranging from air pollution’s influence on the transport and viability of viral particles to its impact on the body’s innate defense mechanisms and long-term immune function (60–62).

The relationship we identified between air pollution and cluster persistence is consistent with several studies in the literature that have reported associations between SARS-CoV-2 infection and COVID-19 severity, and mortality with both short and long-term exposure to air pollution (17, 18, 31). For example, a recent study conducted in Switzerland found an association between long-term exposure to air pollutants and COVID-19 severity and mortality, but only during the first major wave of the pandemic when the national health system was not fully prepared to face the virus (18). However, this study focused exclusively on severity and mortality, while our findings suggest a potential link between air pollution, an increased risk of SARS-CoV-2 infection and prolonged epidemics. In a recent nationwide cohort study in Denmark, Zhang et al. (63) found that individuals facing long-term exposure to air pollution were at an elevated risk of SARS-CoV-2 infection but did not consider the infection dynamics.

Notably, our study presents the advantage of identifying this association consistently over a two-year period and at a very high spatial resolution which reveals that nearby populations may face very unequal risks. This result was confirmed by the models focusing on the Lausanne urban area, suggesting that even within a city, with relatively similar population densities and socioeconomic conditions, local spatial variations in air pollution levels can lead to significant disparities in the spread and persistence of the virus. Furthermore, we also found that flatter epidemics (i.e., lower peakedness) were associated with higher total attack rates. This observation may indicate that areas with higher air pollution levels could be more susceptible to widespread and prolonged outbreaks, further emphasizing the importance of understanding and mitigating the effects of air pollution on public health.

The lagged population density, the location effects, and air pollutants had a major contribution in each period while other predictors only had a slight contribution. Overall, we only identified slight variations in the importance of determinants of cluster persistence across periods indicating stable determinants of SARS-CoV-2 diffusion despite new variant emergence.

Our univariate and multivariate XGBoost models revealed a relatively modest influence of the socioeconomic index on cluster persistence within the study area, indicating that socioeconomic factors may have limited predictive power for this specific aspect of SARS-CoV-2 diffusion dynamics. This outcome contrasts from previous research such as studies (7, 23, 24), which identified a significant relationship between socioeconomic status and COVID-19 outcomes such as case numbers and mortality rates. Notably, our analysis differs in focus: while Sun et al. (23) and Mena et al. (24) investigated case numbers and mortality rates at the local authority district and municipality level, our study examines the persistence of SARS-CoV-2 clustering, offering a perspective on the virus’s spread.

To address the possibility that the SES index’s low importance in our comprehensive model might stem from shared variance with other features, potentially overshadowing its effect, we conducted an additional analysis. A simpler multivariate XGBoost model, structured similarly to those used for air pollutants, was fitted. The results from this streamlined model aligned with our initial findings, further substantiating the SES index’s modest role in predicting cluster persistence. Importantly, our modeling approach prioritizes the practical significance of variables in predicting cluster persistence, rather than their statistical significance. This distinction is key to understanding the nuanced contribution of the socioeconomic index in our analysis. Complementing this, our sensitivity analysis with a Cox PH model, replicating the methodology from our previous work (7), showed a statistically significant association between the SES index and cluster persistence (HR = 0.49, *p* < 0.005), yet the model’s predictive accuracy, as reflected by the C-index of 0.54, remained modest. Other factors such as public health interventions or population behavior may have a more substantial influence. Alternatively, the very low association could be due to limitations in the study design, measurement, or data quality. Further research is needed to confirm these findings and explore the underlying reasons, potentially using alternative measures of socioeconomic status and examining different geographic regions or time periods.

### Strengths and limitations

While previous research has established a link between air pollution and respiratory diseases, including COVID-19, these studies have typically focused on broader regional impacts, often overlooking micro-level variations within small areas. Our findings contribute a novel perspective by revealing significant local spatial variations in the risks associated with air pollution, even within small regions. This granular insight is crucial as it underscores that within a region considered to have overall good air quality, there can still be pockets where air pollution reaches levels that significantly increased the persistence of the virus. These local disparities in air pollution exposure and related health risks highlight the limitations of averaging air quality measures over larger areas, which can mask such critical hotspots of air pollution and associated health risks. The policy implications of these findings suggest that current air quality standards and public health strategies when designed and implemented on a regional basis, may not adequately protect all citizens. Policymakers need to consider implementing finer-scale air quality monitoring systems capable of detecting and addressing these micro-level variations (64). Additionally, it is essential to targeted public health interventions that reflect this fine-scale information, ensuring that preventive measures and resources are specifically allocated according to localized risk levels.

Several methodological strengths of our study include the use of various measures of diffusion dynamics, a long study period (> 2 years), the inclusion of spatial effects, and air pollution data from two different sources (immission model and remote sensing estimation of tropospheric NO_2_). Moreover, our study focuses on a relatively small geographical area with good epidemiological surveillance and presenting diverse sociodemographic and environmental conditions.

Additionally, the methodological approach employing advanced modeling techniques such as XGBoost models and SHapley Additive exPlanations (SHAP) values for model interpretation offered several advantages over traditional spatial methods like spatial lag models or GWR/MGWR (54). The XGBoost allowed us to capture complex non-linear and spatial effects, providing a more comprehensive understanding of the determinants of COVID-19 diffusion dynamics. Moreover, the use of SHAP values enabled a more interpretable and robust assessment of the importance of each feature in our models. SHAP values provided a unified measure of feature importance, considering both the magnitude and direction of the effect, as well as complex interactions between features. This approach made it possible to better understand the contribution of each variable in predicting cluster persistence.

However, our study also shows some limitations. Although we were able to include testing rates in the model, testing bias could still be a concern. The source and place of infection were unavailable, and we could only rely on the place of residence. Additionally, the tropospheric NO_2_ estimation can be subject to biases, which may affect the accuracy of our results for this feature. Lastly, the generalizability of our findings might be limited due to the specific context of our study area.

## Conclusion

Our study highlights the complex spatiotemporal dynamics of COVID-19 diffusion and its association with demographic, socioeconomic and more particularly environmental factors across 2 years of the pandemic. The use of advanced modeling techniques and a wide set of variables allowed us to gain a more detailed understanding of the determinants of COVID-19 spread. Air pollution appears to have played an important role in the COVID-19 pandemic in particular in relation to cluster persistence. Our study underscores thus the importance of implementing effective air quality management strategies to mitigate the potential adverse impacts of pollution on public health, particularly in the context of infectious diseases affecting the upper & lower respiratory tract, like COVID-19.

## Data availability statement

The original contributions presented in the study are included in the article/Supplementary material; further inquiries can be directed to the corresponding author.

## Ethics statement

This study was approved by the Commission cantonale d'éthique de la recherche sur l'être humain (CER-VD), Switzerland. Authorization no. 2020-01302. The studies were conducted in accordance with the local legislation and institutional requirements. The ethics committee/institutional review board waived the requirement of written informed consent for participation from the participants or the participants’ legal guardians/next of kin because given the large number of individuals (± 200,000) involved and the retrospective nature of the study, procuring individual informed consent would pose a substantial challenge.

## Author contributions

DR: Conceptualization, Data curation, Formal analysis, Investigation, Methodology, Visualization, Writing – original draft, Writing – review & editing, Project administration, Validation. AL: Conceptualization, Methodology, Validation, Writing – review & editing. YC: Conceptualization, Validation, Writing – review & editing. DJ: Conceptualization, Validation, Writing – review & editing. SV: Conceptualization, Writing – review & editing. IG: Supervision, Validation, Writing – review & editing. SJ: Conceptualization, Supervision, Validation, Writing – review & editing. GG: Conceptualization, Funding acquisition, Project administration, Supervision, Validation, Writing – review & editing.

## Footnotes

^1^www.microgis.ch
