# Supplementary material for: Environmental and geographical factors influencing the spread of SARS-CoV-2 over 2 years: a fine-scale spatiotemporal analysis
Source: Front Public Health. 2024 Jun 18;12:1298177. doi: 10.3389/fpubh.2024.1298177 (PMC11217542; doi:10.3389/fpubh.2024.1298177)
Supplement: Supplementary file 1 [file Data_Sheet_1.docx]

**Title: Environmental and geographical factors influencing the spread and evolution of SARS-CoV-2 over two years: A fine-scale spatiotemporal analysis**

David De Ridder^1,2,3,4^, Anaïs Ladoy^1,2^, Yangji Choi^5^, Damien Jacot^5^, Séverine Vuilleumier^6^, Idris Guessous*^1,3,4^, Stéphane Joost*^1,2,3^, Gilbert Greub*°^5,7^

# Supplementary material

### Section S1, Hyperparameter optimization

We employed a hyperparameter optimization technique to find the best set of hyperparameters for our XGBoost regression model. The optimization was performed using the Tree-structured Parzen Estimator (TPE) algorithm provided by the Hyperopt library. The TPE algorithm intelligently explores the search space of hyperparameters and converges to the optimal hyperparameters faster compared to other optimization techniques, such as grid search and random search.

The search space for the hyperparameters included: max_depth: The maximum depth of the tree, ranging from 1 to 30 with a step of 2; colsample_bytree: The subsample ratio of columns when constructing each tree, ranging from 0.3 to 1.01 with a step of 0.1; min_child_weight: The minimum sum of instance weight needed in a child, ranging from 1 to 30 with a step of 1; subsample: The subsample ratio of the training instances, ranging from 0.3 to 1.01 with a step of 0.1; learning_rate: The learning rate, ranging from 0.1 to 1.01 with a step of 0.1 and ; gamma: The minimum loss reduction required to make a further partition on a leaf node of the tree, ranging from 0.1 to 5 with a step of 0.2. Finally, the objective function for the optimization was set to regression with squared loss (i.e. reg:squarederror), and the evaluation metric was set to the root mean squared error (RMSE).

To reduce computational cost, we sampled 20% of the input data and performed 5-fold cross-validation to estimate the performance of the model for a given set of hyperparameters. The training data was converted into a DMatrix format for efficient processing with the XGBoost library. The boosting rounds were limited to 500 with early stopping criteria set to 10 rounds, which means that the training would stop if the performance on the validation set did not improve for 10 consecutive rounds.

The optimization procedure was performed with a fixed random state for reproducibility, and the number of evaluations for the TPE algorithm was set as a parameter. The best hyperparameters were obtained by minimizing the RMSE of the model on the test dataset.


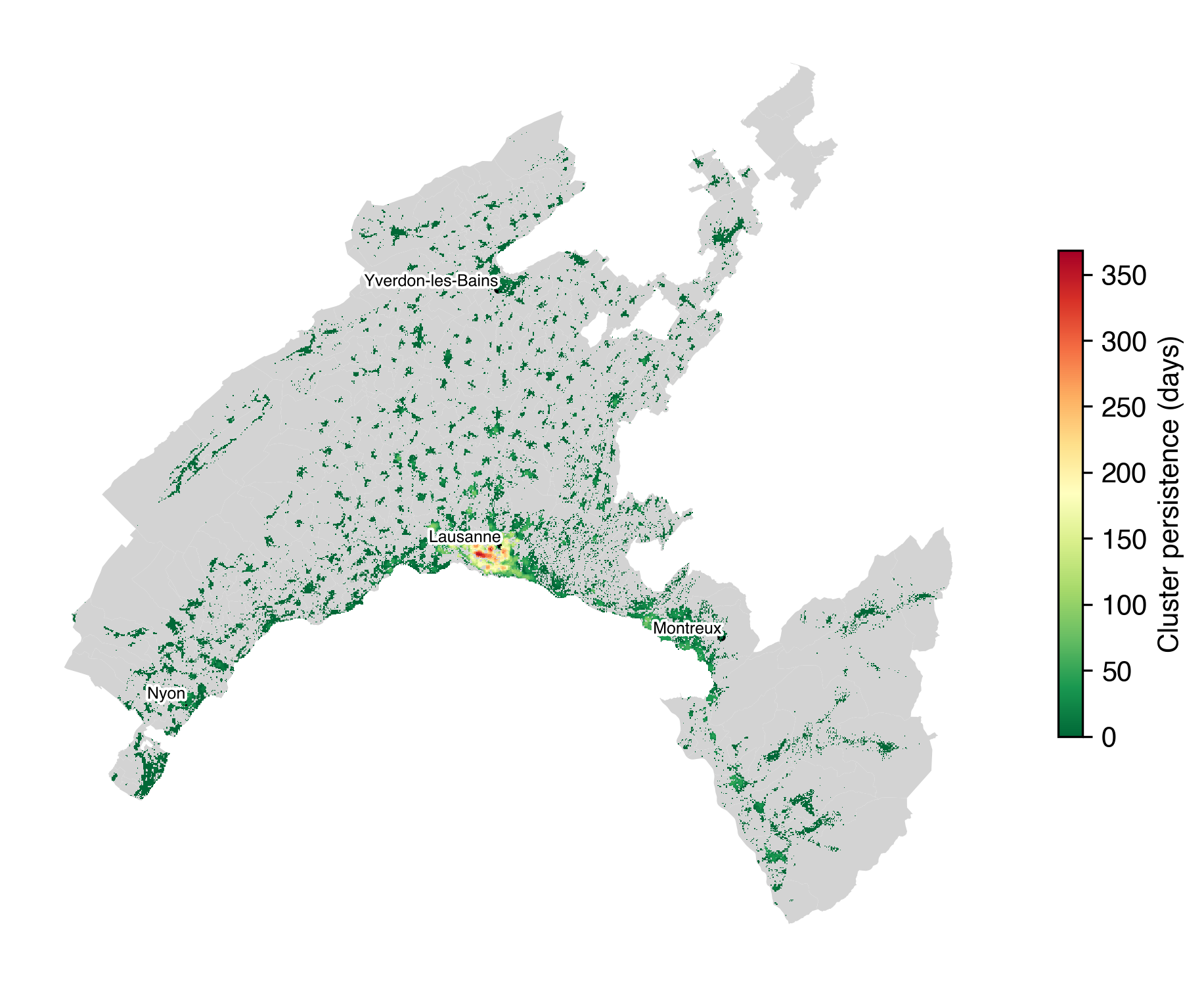


Figure S1 – Spatial distribution of cluster persistence for the whole study period in the canton of Vaud, Switzerland.


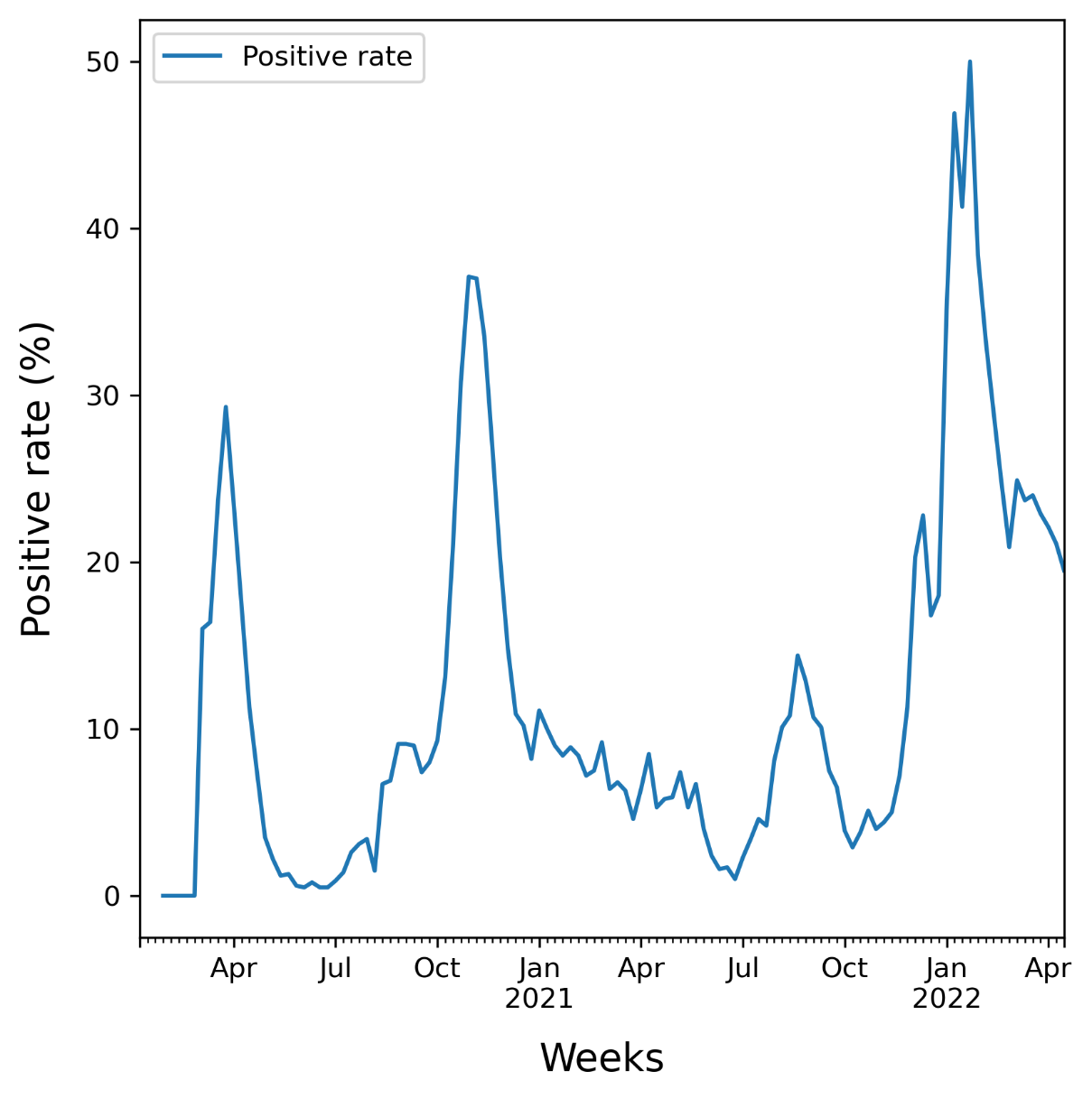


Figure S2 – Time series of the weekly positive rate in the canton of Vaud, Switzerland.


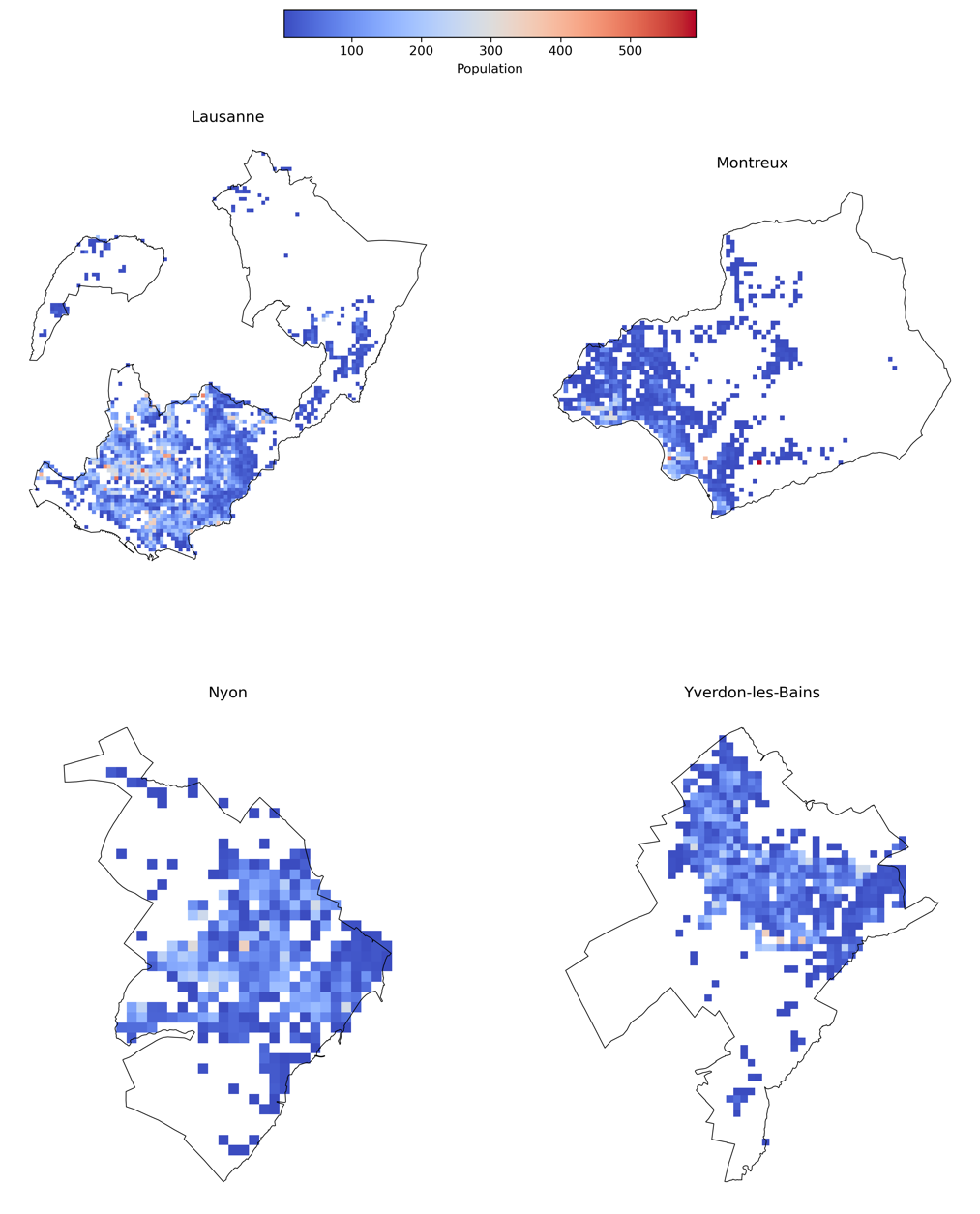


Figure S3 – Examples of population structures for the four most populated municipalities within the study area.


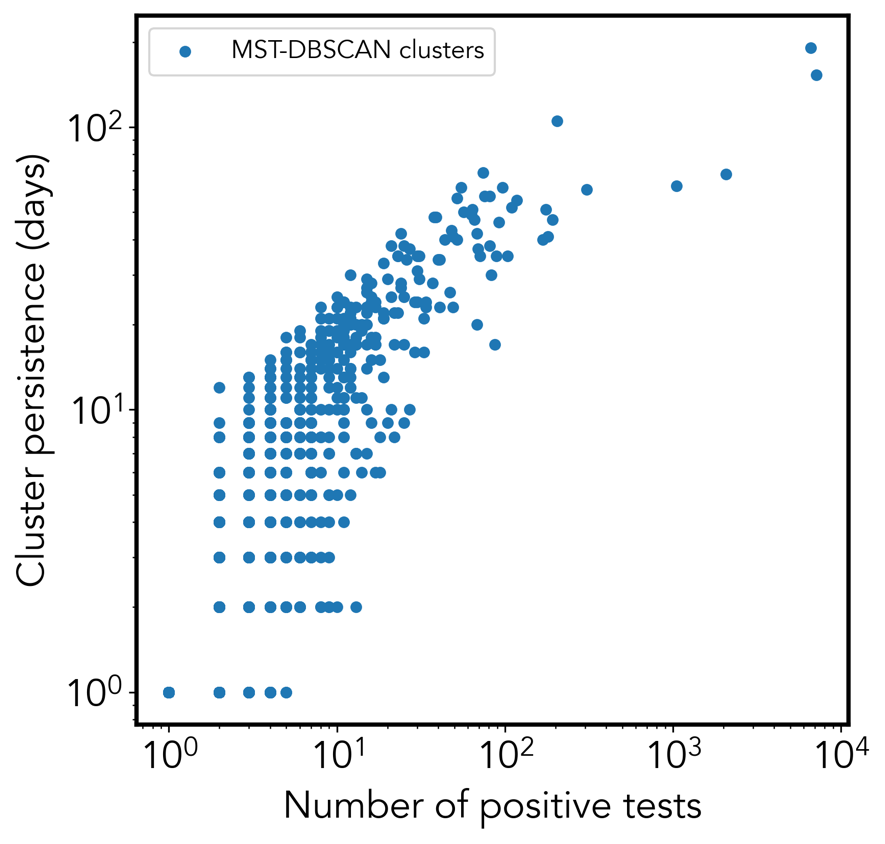


Figure S4 – Relationship between cluster persistence and number of positive RT-PCR tests from cluster emergence to disappearance.


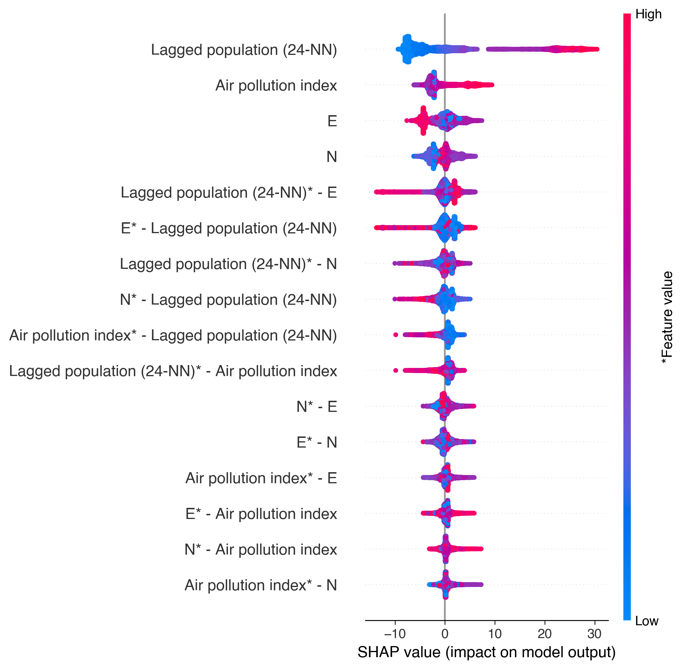

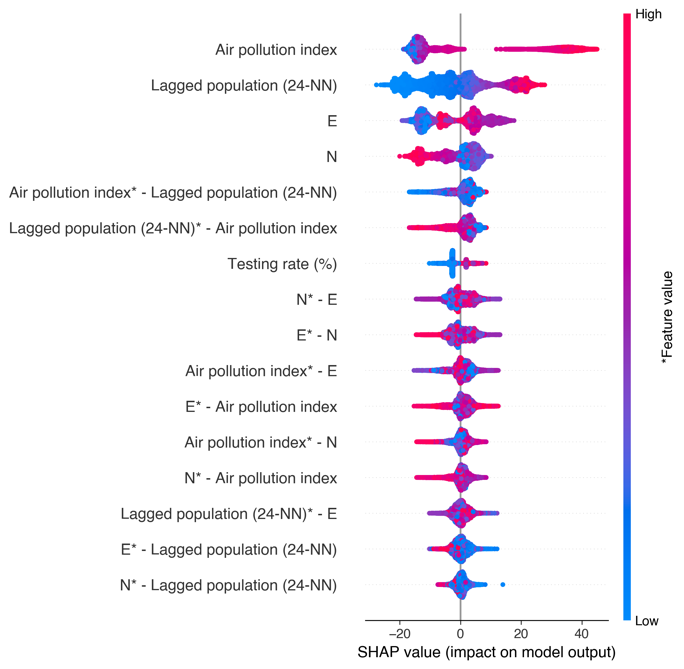


B

A

D

C


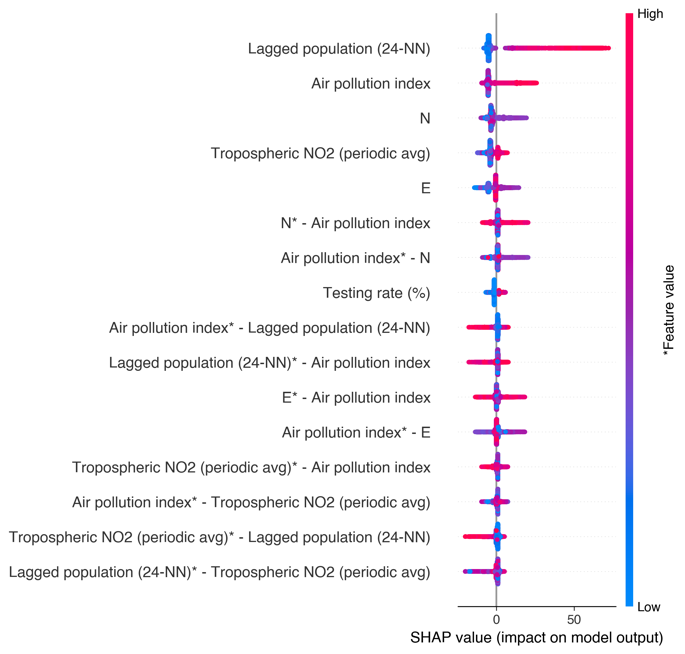

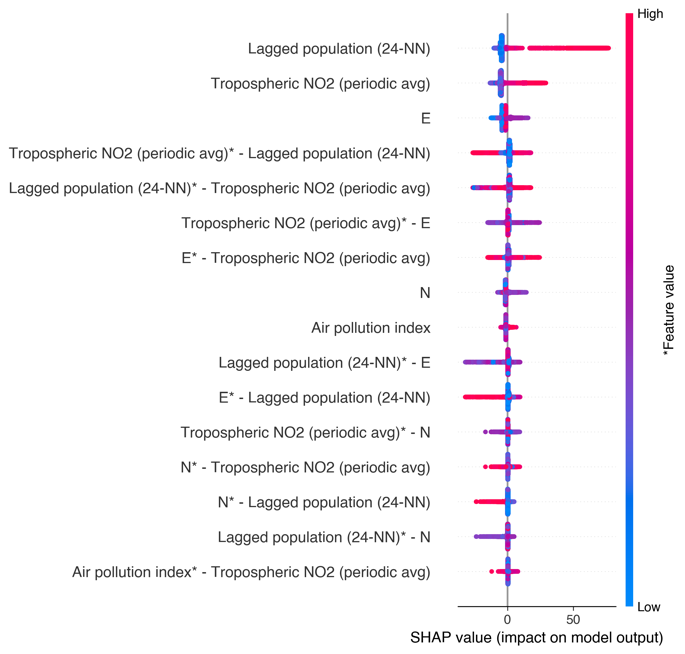


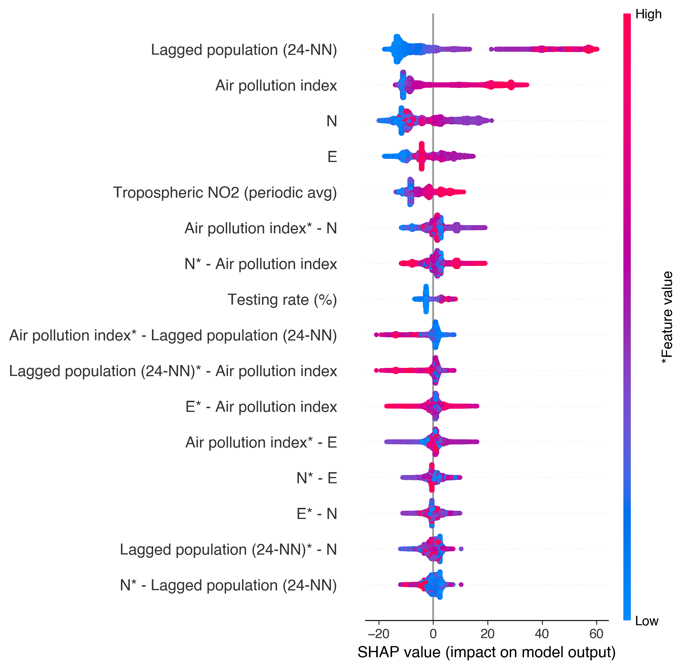


E

Figure S5 – **SHAP summary plots for the cluster persistence XGBoost model by period.** A (Period 1) : lagged population density and the air pollution index have the highest individual SHAP values, indicating a strong influence on cluster persistence; B (Period 2) : influence of NO2 and PM2.5 on cluster duration; C (Period 3) and D (Period 4) : important role of the lagged population density and PM2.5 levels; and E (Period 5): dominant impact of PM2.5 levels and the lagged population density. The direction of the relationships is indicated by the color of the markers; blue markers at the lower side with red markers the higher side indicate a positive relationship while red markers at the lower side with blue markers the higher side indicate a negative relationship.


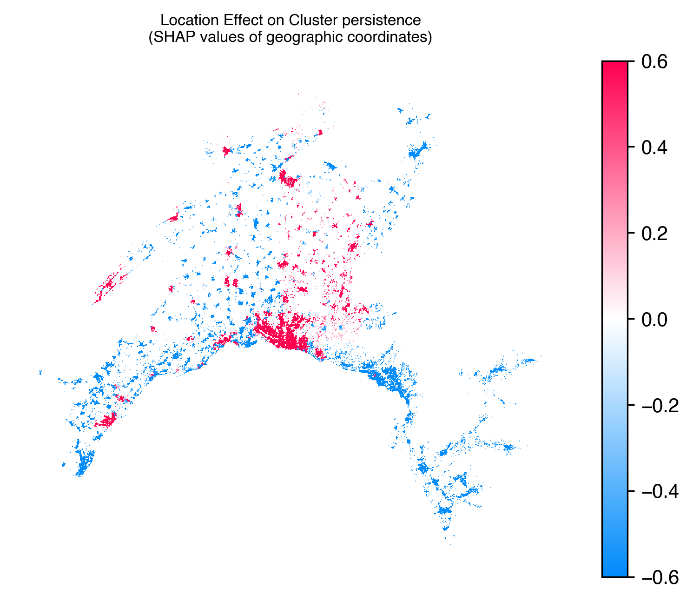

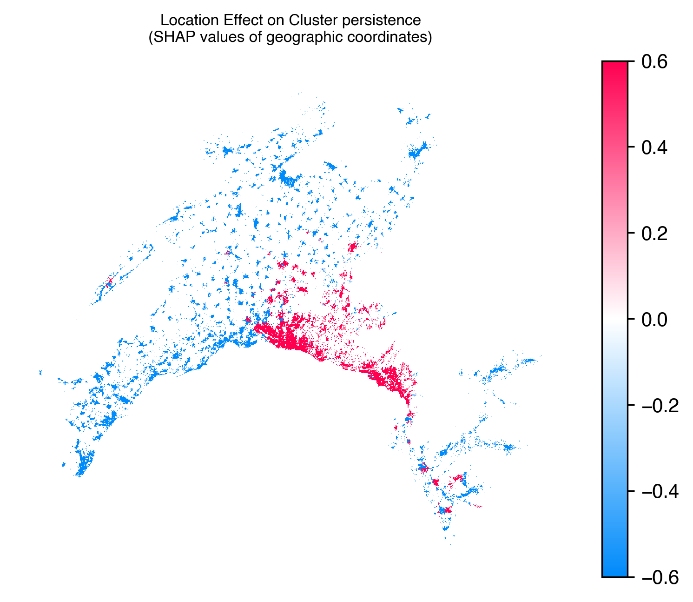


B

A


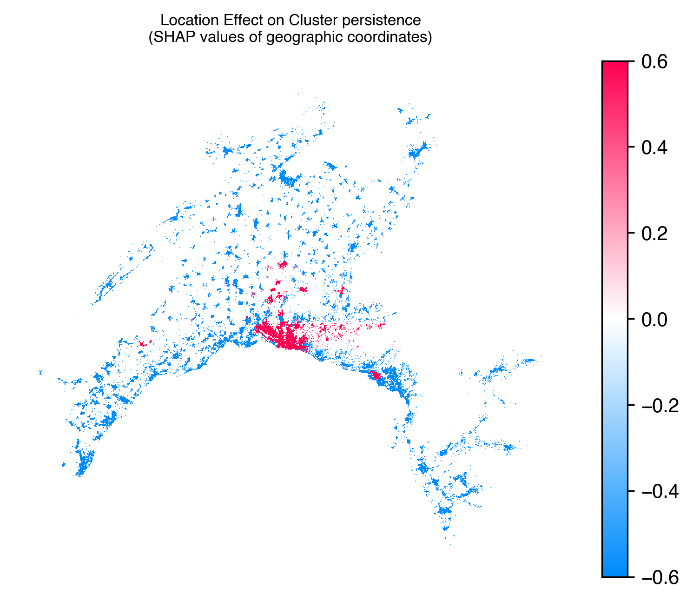

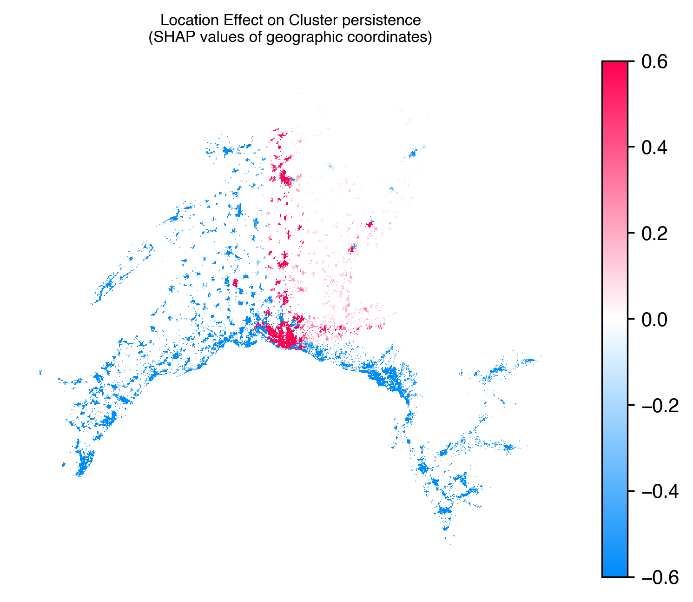


D

C


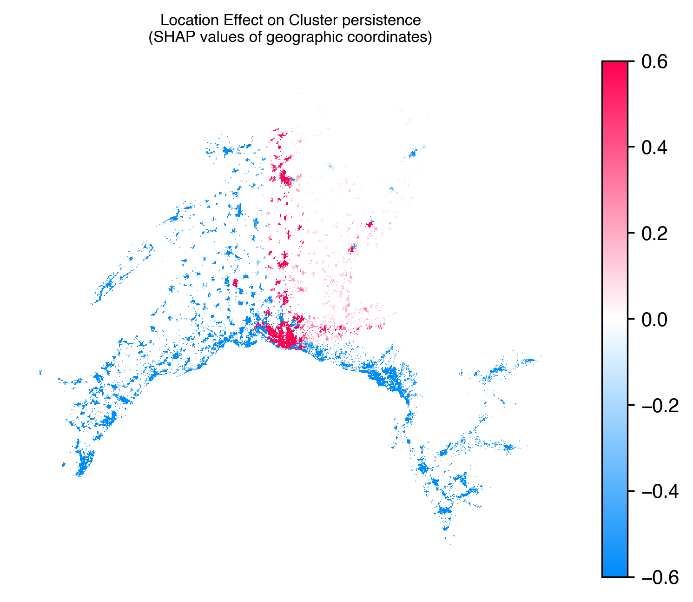


E

Figure S6 – The spatial effect of location on persistence of COVID-19 clusters as measured by the SHAP values of geographic coordinates. A. Period 1, B. Period 2, …, E. Period 5.


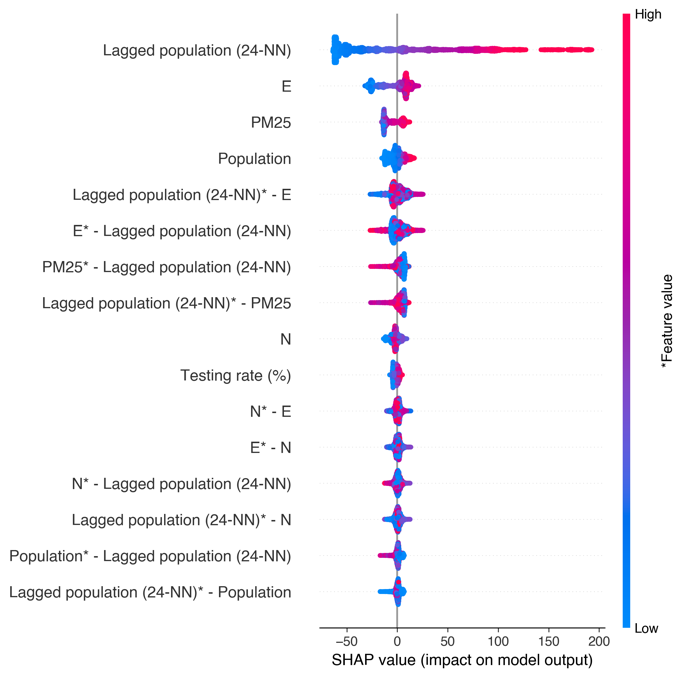

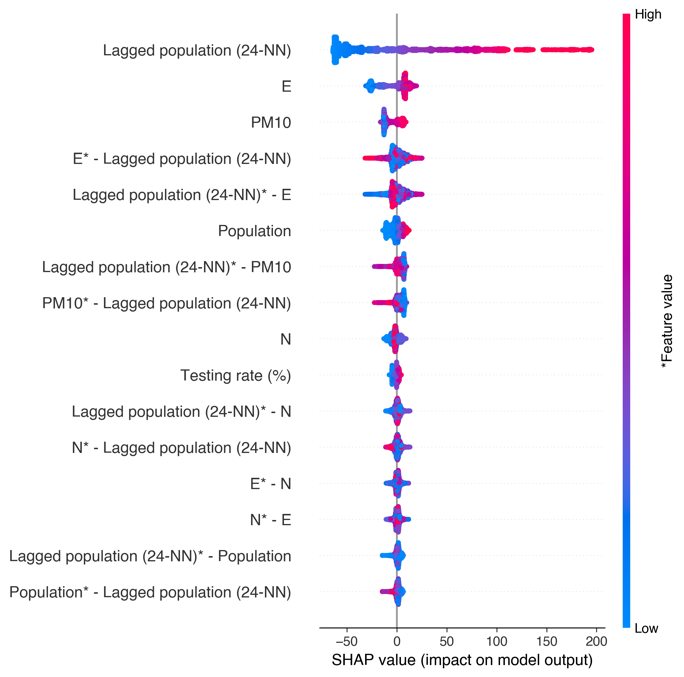


D

C

B

A


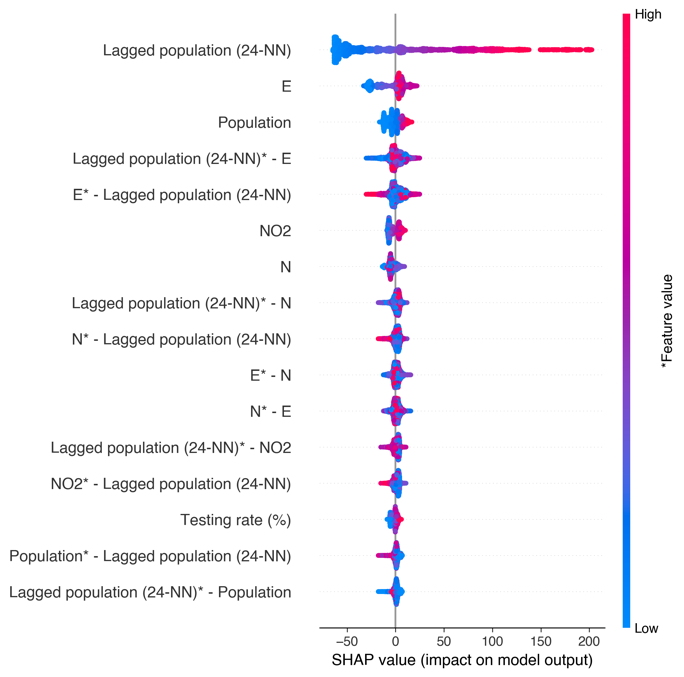

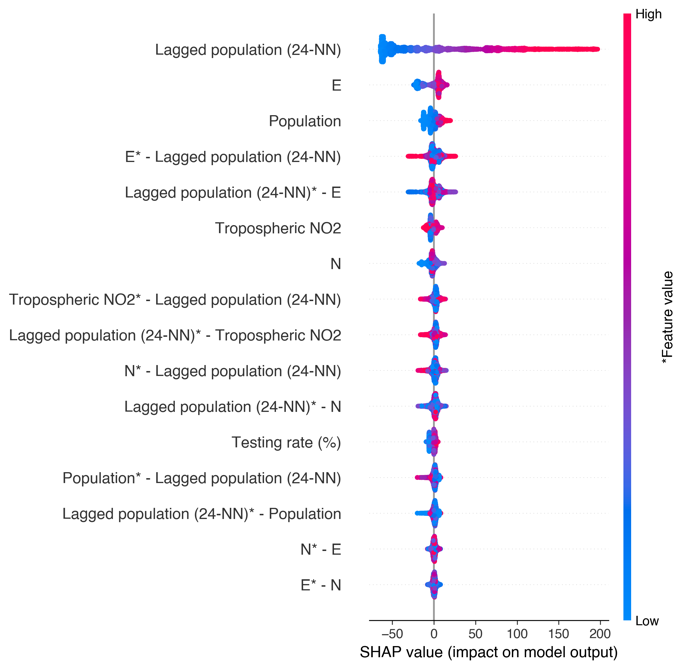


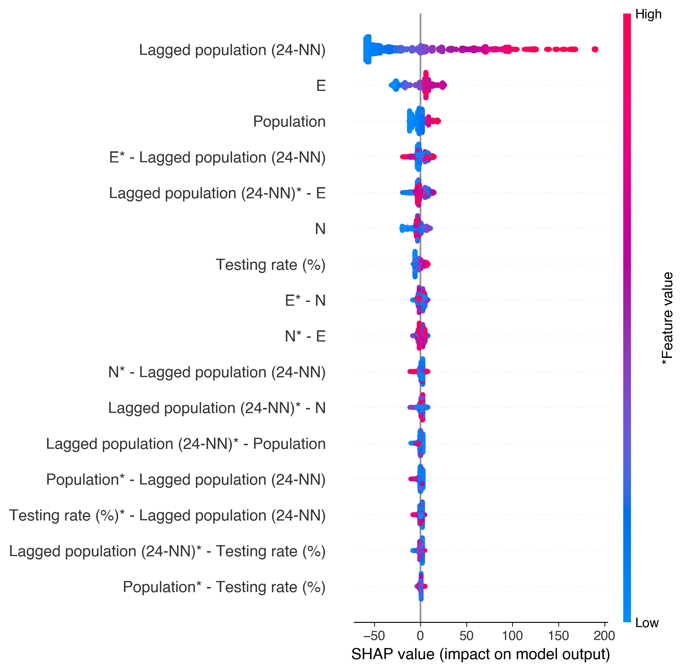


E

Figure S7 – SHAP summary plots of the multivariable XGBoost models fitted for each air pollution feature and the SES index separately. **A.** PM2.5; **B. PM10, C.** NO2; **D.** Tropospheric NO2; and **E.** SES index.

|  | **Period 1** | | **Period 2** | | **Period 3** | | **Period 4** | | **Period 5** | |
| --- | --- | --- | --- | --- | --- | --- | --- | --- | --- | --- |
|  | Air pollution | SES | Air pollution | SES | Air pollution | SES | Air pollution | SES | Air pollution | SES |
| **R^2^** | 0.976 | 0.975 | 0.983 | 0.978 | 0.990 | 0.986 | 0.990 | 0.982 | 0.987 | 0.984 |
| **RMSE** | 2.27 | 2.34 | 4.29 | 4.80 | 2.74 | 3.26 | 2.25 | 3.01 | 4.41 | 5.02 |

Table S1 – **Overall model accuracy of the multivariable XGBoost models.**
